# Multifunctional Fluoropolymer‐Engineered Magnetic Nanoparticles to Facilitate Blood‐Brain Barrier Penetration and Effective Gene Silencing in Medulloblastoma

**DOI:** 10.1002/advs.202401340

**Published:** 2024-04-22

**Authors:** Helen Forgham, Jiayuan Zhu, Xumin Huang, Cheng Zhang, Heather Biggs, Liwei Liu, Yi Cheng Wang, Nicholas Fletcher, James Humphries, Gary Cowin, Karine Mardon, Maria Kavallaris, Kristofer Thurecht, Thomas P. Davis, Ruirui Qiao

**Affiliations:** ^1^ Australian Institute of Bioengineering & Nanotechnology The University of Queensland St Lucia Queensland 4072 Australia; ^2^ National Imaging Facility Centre for Advanced Imaging The University of Queensland St Lucia Queensland 4072 Australia; ^3^ ARC Training Centre for Innovation in Biomedical Imaging Technology The University of Queensland St Lucia Queensland 4072 Australia; ^4^ Children's Cancer Institute Lowy Cancer Research Centre UNSW Sydney Kensington New South Wales 2052 Australia; ^5^ School of Clinical Medicine Faculty of Medicine & Health UNSW Sydney Kensington New South Wales 2052 Australia; ^6^ UNSW Australian Centre for Nanomedicine Faculty of Engineering UNSW Sydney Kensington New South Wales 2052 Australia; ^7^ UNSW RNA Institute Faculty of Science UNSW Sydney Kensington New South Wales 2052 Australia

**Keywords:** blood‐brain barrier, medulloblastoma, nanoparticles, siRNA, theranostics

## Abstract

Patients with brain cancers including medulloblastoma lack treatments that are effective long‐term and without side effects. In this study, a multifunctional fluoropolymer‐engineered iron oxide nanoparticle gene‐therapeutic platform is presented to overcome these challenges. The fluoropolymers are designed and synthesized to incorporate various properties including robust anchoring moieties for efficient surface coating, cationic components to facilitate short interference RNA (siRNA) binding, and a fluorinated tail to ensure stability in serum. The blood‐brain barrier (BBB) tailored system demonstrates enhanced BBB penetration, facilitates delivery of functionally active siRNA to medulloblastoma cells, and delivers a significant, almost complete block in protein expression within an in vitro extracellular acidic environment (pH 6.7) – as favored by most cancer cells. In vivo, it effectively crosses an intact BBB, provides contrast for magnetic resonance imaging (MRI), and delivers siRNA capable of slowing tumor growth without causing signs of toxicity – meaning it possesses a safe theranostic function. The pioneering methodology applied shows significant promise in the advancement of brain and tumor microenvironment‐focused MRI‐siRNA theranostics for the better treatment and diagnosis of medulloblastoma.

## Graphical abstract

Medulloblastoma is the most common pediatric brain malignancy. Here, multifunctional theranostic nanoparticles capable of 1)blood‐brain barrier penetration, 2) contrast enhancement of brain tumors (magnetic resonance imaging), and 3) superb short interference RNA delivery is presented in vitro and in vivo (orthotopic mouse model). The nanotechnology applied here holds significant promise not just in treating medulloblastoma, but all brain cancers.

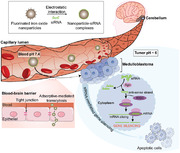

## 1 Introduction

Medulloblastoma is the most diagnosed malignant form of brain cancer in children.^[^ ^1^ ^]^ Although improvements in treatment over the last few decades have helped to increase survival, medulloblastoma and other brain cancer subtypes collectively take the lives of more children and adults under the age of 40 than any other cancer. In 2020 alone, the global burden of brain cancers resulted in 251 329 deaths.^[^ ^2^ ^]^ Additionally, the side effects of brain cancer treatment often cause long‐term damage and elicit a poor quality of life.^[^ ^3^ ^]^ The blood‐brain barrier (BBB) is the physiological cause of why patient outcomes are so poor. This highly selective and protective vascular channel prevents access to many of the best chemotherapeutics used for other non‐brain‐related cancer types.^[^ ^4^ ^]^ Even when chemotherapeutics do cross, poor bioavailability results in the need for high‐dose exposure – with harsh side effects the consequence. Additionally, size restrictions on crossing prevent the use of alternative protein‐based treatments such as recombinant proteins and therapeutic antibodies.^[^ ^5^ ^]^ Non‐viral nanoparticles are engineered delivery vehicles that can facilitate better passage across the BBB.^[^ ^4^ ^]^ They can be used to deliver chemotherapeutics as well as newer targeted approaches including gene‐based therapeutic strategies including short interference RNA (siRNA).

Nanoparticle delivery of siRNA therapeutics enables the silencing of oncogenic regulators of brain cancers, initiating targeted cancer cell death.^[^ ^6^ ^]^ However, when healthy cells are exposed to the siRNA no lasting effects are detected.^[^ ^7^ ^]^ The rationale for their use in medulloblastoma is underpinned by limited reports of side effects, good bio ability affect, and potential to target brain cancer oncogenes with high specificity. Last, nanoparticle‐siRNA facilitates better tumor uptake through the enhanced permeability and retention effect.^[^ ^7^ ^]^ A current impediment affecting translation is ineffective nanoparticle‐facilitated carriage in the blood, restricted passage across the BBB and tumor vasculature; and more specifically, lack of functional activation (gene silencing) in target cells located within the complex acidic tumor microenvironment.^[^ ^8^ ^]^

In our prior research efforts, we successfully developed rapid and dependable techniques for creating hybrid nanomaterials that combine organic and inorganic components, specifically polymer and iron oxide.^[^ ^9^ ^]^ These hybrid material nanoparticles exhibited remarkable stability and a wide range of functions that cater to various biological applications including drug delivery.^[^ ^9^, ^10^ ^]^ In this current study, we have extended our work to try to address the key challenges faced by siRNA therapeutic targeted toward medulloblastoma by synthesizing innovative functional fluoropolymers crafted to modify the surface of magnetic iron oxide nanoparticles (IONPs). Our aims for these IONPs were to facilitate greater BBB penetration, enhance siRNA delivery, and simultaneously leverage the inherent characteristics of iron oxide as a contrast agent for magnetic resonance imaging (MRI), enabling the tracking of uptake in cancer cells and siRNA gene silencing effects. To achieve these aims, we employed the reversible addition‐fragmentation chain transfer (RAFT) polymerization technique, which enabled us to create multifunctional fluoropolymers with several advantageous attributes. These attributes encompass robust binding to the IONP surface, incorporation of cationic groups for effective siRNA binding, introduction of fluorinated segments to shield siRNA from degradation in serum,^[^ ^11^ ^]^ and incorporation of features that trigger release in the acidic tumor microenvironment to facilitate targeted delivery and effective gene silencing.

**Scheme 1:**
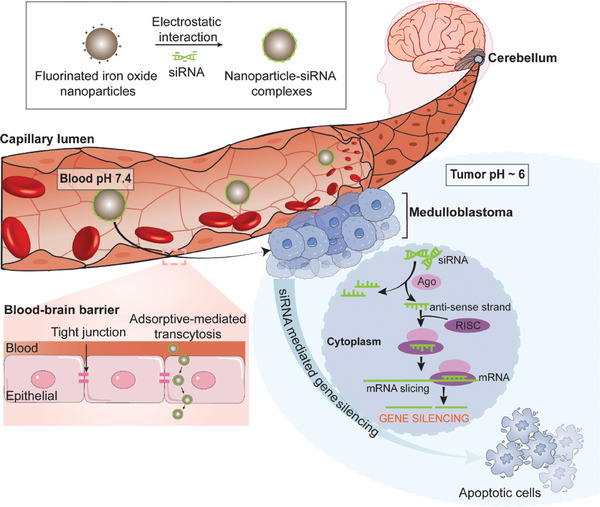
Fluorinated iron oxide nanoparticles facilitate passive crossing of the blood‐brain barrier and delivery of siRNA into medulloblastoma cells.

To this effect, we developed two original perfluoropolyether (PFPE) polymer‐engineered iron oxide nanoparticle platforms and established that unique surface topology as a result of the different orientation of monomer composition influenced particle size, charge, siRNA binding and cellular uptake, and gene silencing efficiency. Intriguingly, our investigations revealed that the fluoropolymers grafted IONPs, particularly the formulation incorporating siRNA binding, exhibited a remarkable capacity to induce gene silencing in medulloblastoma models (**Scheme** **1**). This success could be attributed to the exceptionally effective delivery facilitated by these nanoparticles. Furthermore, our work showcased the capability of PFPE‐based fluoropolymers to facilitate transportation across the BBB, thereby enhancing the cellular uptake of IONPs‐siRNA assemblies. The introduction of the cationic polymer component, alongside the fluoropolymer, introduced a pH‐responsive mechanism for siRNA release within the tumor microenvironment. Additionally, we determined that circulatory times and biological clearance mechanisms were affected by both the incorporation of fluorine and the surface topology of the fluorinated hybrids in vivo. Finally, we demonstrate fluorinated IONP as an effective theranostic platform for MRI imaging and therapeutic siRNA delivery for medulloblastoma by using in vivo orthotopic models.

## 2 Results

### 2.1 Synthesis of Fluoropolymer

**Figure 1:**
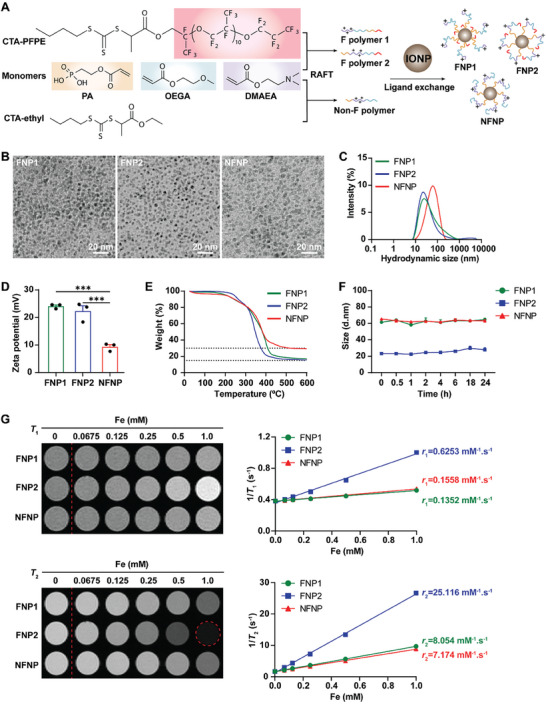
Preparation and characterization of polymer‐coated IONPs. A) Scheme of synthesizing polymer‐coated nanoparticles. B) Representative TEM images for FNP1, FNP2, and NFNP (scale bar: 20 nm). C) Graph illustrating hydrodynamic size distribution of FNP1, FNP2, and NFNP in H_2_O. D) Graph depicting the Zeta potential in H_2_O, ^***^ *p* ≤ 0.001, one‐way ANOVA, Tukey's multiple comparisons test. E) Graph showing results of TGA analysis. F) Colloidal stability in PBS for up to 24 h, the size distribution was analyzed by intensity. G) Representative image demonstrating *T*_1_ and *T*_2_ relaxation rate (*r*_1_ and *r*_2_) of FNP1, FNP2, and NFNP in OptiMem against the concentration of Fe ion determined by 7.0 T MRI and quantified results as graphed data. All experiments described are n = 3.

A series of fluorinated polymers containing different functionalized segments were synthesized via RAFT polymerization as depicted in **Figure** **1**A. High‐fluorine containing hydroxy‐terminated perfluoropolyether (PFPE‐OH) with excellent biocompatibility^[^ ^12^ ^]^ was used to prepare a macro RAFT chain transfer agent (CTA‐PFPE) via 1‐Ethyl‐3‐(3‐dimethylaminopropyl)carbodiimide (EDC) coupling and confirmed by ^1^H NMR and ^19^F NMR (Figures S1, S2, Supporting Information). Extensive evidence supports the critical role of the primary structures of polymers, particularly the distribution of monomer sequences, in shaping the topology, morphology, and size, as well as the dynamic and physical properties of their self‐assemblies.^[^ ^13^ ^]^ Thus, to investigate the impact of different self‐assembled folding on the topology and physiochemical properties of polymers, two fluoropolymers with reversed polymerization sequences and a non‐fluorinated polymer as the control group were prepared. Monophosphonate monomers were synthesized and polymerized to obtain PFPE‐poly(2‐(dimethoxyphosphoryl)ethyl acrylate)_6_ (denoted as PFPE‐P(PA)_6_), endowing the fluoropolymers with a high binding affinity to IONPs surface.^[^ ^10^, ^14^ ^]^ The degree of polymerization (DP) for the monophosphonate monomer was calculated as 6, which we calculated by comparing the integral of the peak (b) at 4.0‐4.5 ppm belonging to the methylene group next to the monophosphonate monomer ester bond and the peak (a) at 0.95 ppm belonging to the methyl group of CTA (Figure S3, Supporting Information). The PFPE‐P(PA)_6_ was used as a macro‐CTA for a second polymerization step in order to incorporate dimethylaminoethyl acrylate (DMAEA) and oligoethylene glycol acrylate (OEGA) monomers, leading to the production of PFPE‐P(PA_6_‐*b*‐(OEGA_12_‐*co*‐DMAEA_6_)) – confirmed by ^1^H NMR (Figure S4, Supporting Information). Notably, DMAEA, with a pKa ≈ 8.41, is a widely adopted monomer for the preparation of branched polymers,^[^ ^15^ ^]^ which can be used as a comparable alternative to polyethylenimine (PEI) for complexing siRNA.^[^ ^16^ ^]^ We prepared two additional polymers, namely PFPE‐P((OEGA_11_‐*co*‐DMAEA_6_)‐*b*‐PA_5_) and BTPA‐P((OEGA_11_‐*co*‐DMAEA_5_)‐*b*‐PA_8_), through reversed polymerization sequences using CTA‐PFPE and non‐fluorinated CTA, respectively (Figure 1A). All synthesized polymers showed well‐resolved peaks in their ^1^H NMR spectra, with a successful assignment of these peaks to their corresponding protons (Figures S5–S8, Supporting Information). The polydispersity (*Ð*) and molecular weight of synthesized polymers were characterized by size exclusion chromatography (SEC) and summarized in Table S1 and Figure S9 (Supporting Information).

### 2.2 Preparation and Physicochemical Characterizations of Polymer‐Iron Oxide Nanoparticles

Exceedingly small iron oxide nanoparticles (ESIONPs) – size below 5 nm were synthesized via the well‐established thermal decomposition method.^[^ ^17^ ^]^ However, here we doubled the concentration of the surface capping ligand (oleyl alcohol), thereby obtaining highly dispersed magnetic nanoparticles. Although ESIONPs have enormous potential, stabilizing nanoparticles that are extremely small in size is always challenging because of the high surface area to volume ratio, which makes them more prone to aggregation.^[^ ^18^ ^]^ Transmission electron microscopy (TEM) analysis showed that the ESIONPs we synthesized had a uniform size distribution and average size of 3.30 ± 0.51 nm (Figure S10A, Supporting Information). The surface of the ESIONPs was capped by the organic ligand oleic acid (OA), evidenced by the FT‐IR spectra (Figure S11, Supporting Information).

Hybrid nanoparticles were synthesized through a precisely controlled ligand exchange approach, involving the grafting of specific multidentate phosphonic acid‐containing polymers onto the surface of ESIONPs.^[^ ^14^, ^19^ ^]^ Consequently, we successfully obtained two fluorinated IONPs coated with PFPE‐P(PA_6_‐*b*‐(OEGA_12_‐*co*‐DMAEA_6_)) (FNP1) and PFPE‐((OEGA_11_‐*co*‐DMAEA_6_)‐*b*‐PA_5_) (FNP2), respectively. For control purposes, we generated non‐fluorinated nanoparticles (NFNP) by employing the BTPA‐P((OEGA_11_‐*co*‐DMAEA_5_)‐*b*‐PA_8_) (Figure 1A). TEM analysis showed that FNP1, FNP2, and NFNP in H_2_O were monodispersed, uniform in shape, and without any visible aggregation (Figure 1B; Figure S10, Supporting Information). The hydrodynamic size was analyzed using dynamic light scattering (DLS) by intensity (FNP1: 69.20 ± 6.57, FNP2: 45.67 ± 3.40, NFNP: 65.36 ± 1.34 nm) (Figure 1C). Size differences between FNP1 and FNP2 were observed when measuring intensity and zeta average, which we attributed to the outer PFPE hydrophobic layer of FNP2. DLS analysis of the zeta potential confirmed FNP1, FNP2, and NFNP all displayed a positively charged polymer coating, and therefore were potential candidates for siRNA delivery through established electrostatic interactions. Notably, the two fluoropolymer‐decorated nanohybrids exhibited a significantly higher positive charge (*p* ≤ 0.001) than their non‐fluoropolymer counterpart (Figure 1D). To understand why, we examined the grafting density of the FNP1, FNP2, and NFNP nanohybrids using thermogravimetric analysis (TGA). The results revealed that FNP1 and FNP2 exhibited a ≈15% higher polymer content compared to NFNP (Figure 1E), which explained the higher positive charge observed in the fluoropolymer‐decorated nanohybrids. Specifically, the TGA analysis suggests that the >10 wt.% of fluorine content in the fluoropolymer endowed the nanohybrids with a lower surface energy and higher hydrophobicity, which together, acted synergistically with multidentate phosphonic acids to encourage a superior grafting density of the polymers onto the 3 nm IONPs.^[^ ^20^ ^]^ We next examined the colloidal stability of FNP1, FNP2, and NFNP over a 24 h period following immersion in phosphate‐buffered saline (PBS). Even though all of the three nanoparticles were stable over 24 h, the DLS data determined significant variability in size for FNP2 in PBS (≈20 nm) compared to that in H_2_O (≈40 nm), suggesting that FNP2 could be affected by salts in a biologically relevant medium (Figure 1F).

### 2.3 Fluorinated and Non‐Fluorinated Polymer‐Iron Oxide Nanoparticles Provide Contrast for MRI

The ability of FNP1, FNP2, and NFNP to be an effective contrast agent for MRI was verified in OptiMem using the 7.0 Tesla (7.0 T) Bruker ClinScan scanner. As shown in Figure 1G, signal intensities were increased proportionally to the Fe concentration for all three nanoparticles. In contrast to the bright signals observed in the *T*_1_‐weighted image, darker signals were detected with increasing concentration for all three nanoparticles in *T*_2_‐weighted images. FNP1, FNP2 and NFNP exhibited longitudinal relaxivities (*r*_1_) of 0.1352, 0.6253, and 0.1558 mM^−1^ s^−1^, respectively, with transverse relaxivities (*r*_2_) of 8.054, 25.116, and 7.174 mM^−1^ s^−1^ (Table S2, Supporting Information). Meanwhile, the critical parameter of *r*_2_/*r*_1_ is pivotal in discerning the suitability of materials as either *T*_2_ or *T*_1_ contrast agents. Typically, a ratio of *r*_2_/*r*_1_ ≥ 10 is deemed conducive for *T*_2_ contrast agents, whereas a ratio of *r*_2_/*r*_1_ < 2 is considered preferable for *T*_1_ contrast agents.^[^ ^21^ ^]^ As shown in Table S2 (Supporting Information), the iron oxide content of all three nanoparticles resulted in *r*_2_/*r*_1_ ratio ≥10, therefore FNP1, FNP2, and NFNP can all behave as *T*_2_ contrast agents. The 9.4 T MRI results on FNP1, FNP2, and NFNP in H_2_O are shown in Figure S12 (Supporting Information), and also demonstrate *r*_2_/*r*_1_ ratio ≥10 (Table S3, Supporting Information).

### 2.4 Fluoropolymer Engineered IONPs Provide Enhanced Stability for siRNA

**Figure 2:**
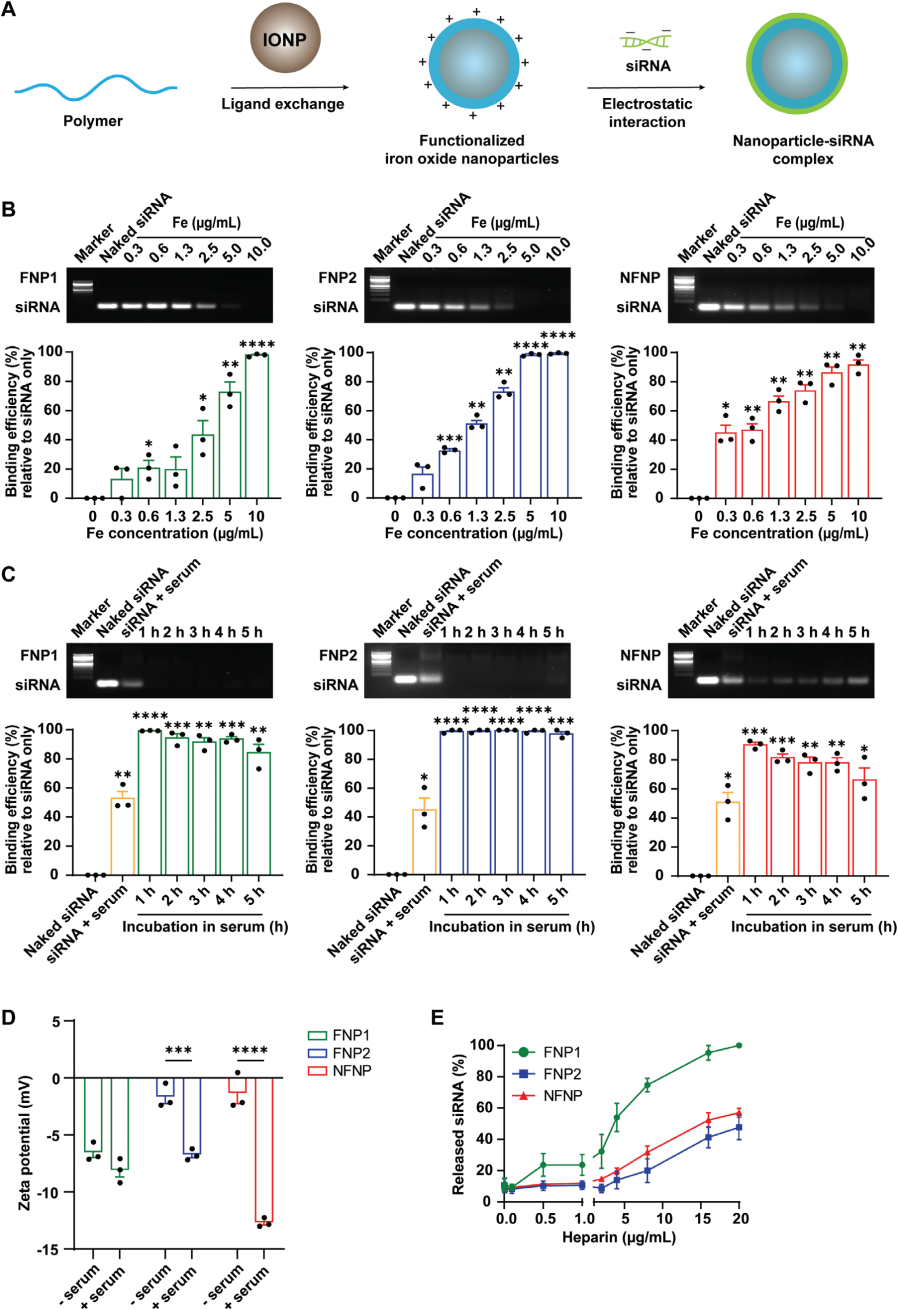
siRNA binding affinity and nanoparticle protection against serum degradation over time. A) Scheme for the formation of NP‐siRNA complexes. B) Representative electrophoresis gel images and corresponding densitometry graphs (FNP1: green, FNP2: blue, NFNP: red) illustrate nanoparticle binding affinity to 25 nM siRNA in H_2_O at an escalating dose of nanoparticles (based on weight of Fe in NP‐siRNA complexes), ^*^ *p* ≤ 0.05, ^**^ *p* ≤ 0.01, ^***^ *p* ≤ 0.001, ^****^ *p* ≤ 0.0001, one sample t and Wilcoxon test. Naked siRNA served as control. C) Representative electrophoresis gel images and corresponding densitometry graphs (FNP1: green, FNP2: blue, NFNP: red) demonstrate the stability of NP‐siRNA complexes of 10 µg mL^−1^ incubated in DMEM/10% FBS up to 5 h, ^*^ *p* ≤ 0.05, ^**^ *p* ≤ 0.01, ^***^ *p* ≤ 0.001, ^****^ *p* ≤ 0.0001, one sample *t* and Wilcoxon test. Naked siRNA served as negative control, and siRNA in serum served as positive control. D) Graph depicting the zeta potential in DMEM with and without serum, ^***^ *p* ≤ 0.001, ^****^ *p* ≤ 0.0001, two‐way ANOVA, Šídák's multiple comparisons test. E) Graph depicting binding affinity of NP‐siRNA complexes when incubating with increasing concentrations of heparin (0–20 µg mL^−1^). All experiments described are n = 3.

Non‐targeting siRNA were attached to the fluoropolymer and non‐fluoropolymer‐engineered IONPs (FNP1, FNP2, and NFNP) through electrostatic interaction to form the NP‐siRNA complex (**Figure** **2**A). To establish the binding potential of FNP1, FNP2, and NFNP, escalating concentrations of nanoparticles based on the weight of iron (Fe) were complexed to 25 nM siRNA. All test nanoparticle formulations fully bound free siRNA at 10 µg Fe (*p* ≤ 0.0001, *p* ≤ 0.0001, *p* ≤ 0.01, respectively) (Figure 2B). To determine any differences in the ability of the three different nanoparticle formulations to protect siRNA from RNAses and enzyme degradation akin to what would occur in the blood, nanoparticle‐siRNA complexes were exposed to 10% serum proteins over a period of 5 h (Figure 2C). Notably, FNP1 and FNP2 were more effective at protecting siRNA from degradation than NFNP (≈88% *p* ≤ 0.01, ≈98% *p* ≤ 0.001, ≈68% *p* ≤ 0.05, respectively). Given the data obtained from the TGA analysis, we attribute the better protection from the lower surface energy, higher hydrophobicity, and overall improved grafting density of the fluorinated polymer hybrid.

Studies have previously demonstrated that nanoparticles together with their bound cargo should be 15–100 nm – with a near neutral‐slightly positive charge if they are to affectively cross the BBB.^[^ ^22^ ^]^ Given that the binding of siRNA may significantly change the hydrodynamic size of polymer nanoparticles, we monitored the size of FNP1, FNP2, and NFNP before (69.20 ± 6.57, 45.67 ± 3.40, and 65.36 ± 1.34 nm) and after complexation to siRNA.^[^ ^23^ ^]^ The hydrodynamic size of FNP1, FNP2, and NFNP were significantly changed following complexations (100.69 ± 3.90, 61.25 ± 2.82, and 44.81 ± 0.09 nm, respectively) (Figure S13A, Supporting Information). We next measured the zeta potential, and as predicted we did identify a negative shift in charge following binding of the siRNA. After 20 min of incubation, the zeta potential of FNP1 transitioned from ≈+24 to –6.6, FNP2 from ≈+22 to –1.7, and NFNP from ≈+9.3 to –1.4 mV, respectively.

We next sought to determine whether fluorine would reduce protein adsorption when the complexes were exposed to 10% serum – identifiable by a potential increase in size and more negative zeta potential. FNP1 and NFNP‐siRNA complexes showed significant change in their hydrodynamic size, whilst FNP2‐siRNA did not significantly change (Figure S13B, Supporting Information). When we examined the zeta potential following exposure to serum proteins (10%), we observed an additional reduction in zeta potential in the three nanohybrids. However, the greatest shift was observed in the NFNP‐siRNA (–1.4 to –12.7 mV), suggesting that the addition of fluorine during polymer synthesis may either help reduce protein adsorption or change the proteins preferentially absorbed, and thus, be a method that positively impacts biodistributions (Figure 2D).

In order for siRNA to bind target intracellular mRNA it must be released by the delivery vehicle inside of the cytoplasm undamaged. However, if the binding affinity between the siRNA and nanoparticle is too strong, this will significantly impact the release of functional siRNA.^[^ ^24^ ^]^ To investigate the binding affinity between IONPs and siRNA, we incubated FNP1‐siRNA, FNP2‐siRNA, and NFNP‐siRNA with the intracellular anionic competitive molecule heparin in an attempt to displace the siRNA.^[^ ^25^ ^]^ For these experiments, we used heparin at increasing concentrations (0, 0.01, 0.1, 0.5, 1.0, 2.0, 4.0, 8.0, 16.0, and 20.0 µg mL^−1^). The results demonstrated that at all heparin concentrations, FNP1 was more amenable to disassociation in the presence of heparin than either FNP2 or NFNP (Figure 2E).

### 2.5 Fluorine Plays a Bio‐Proofing Role for IONPs and Improves siRNA Delivery In Vitro

**Figure 3:**
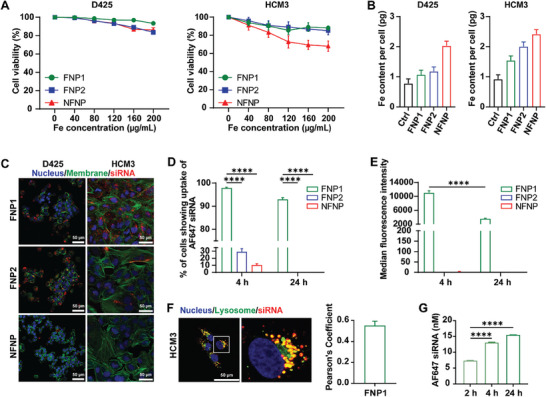
Cytotoxicity of nanoparticle vehicles and cellular uptake of NP‐siRNA complexes. A) Graphs illustrating the cytotoxic effect of nanoparticle vehicles on D425 and HCM3 respectively. B) Iron content determination after transfection with nanoparticles on D425 and HCM3 using ICP‐OES. C) Representative confocal microscopy images showing cellular uptake of 100 nM siRNA (red) complexed to 100 µg mL^−1^ NP at 4 h post‐transfection in D425 and HCM3 cells, respectively (scale bar: 50 µm). D) and E) Graphed flow cytometry data showing quantitative analysis of siRNA uptake in D425 as a percentage of cells D) ^****^ *p* ≤ 0.0001, two‐way ANOVA, Dunnett's multiple comparisons tests, and median fluorescence intensity E) ^****^ *p* ≤ 0.0001, two‐way ANOVA, Šídák's multiple comparisons test at 4 and 24 h post‐transfection. F) Representative live cell confocal microscopy images showing the colocalization between lysosomes (green) stained by lysotracker and siRNA (red) trafficking delivered by FNP1 in HCM3 cells (scale bar: 50 µm). G) Exocytosis of AF647 siRNA at 2, 4, and 24 h post‐transfection on hCMEC/D3 cell line (FNP1: 200 µg mL^−1^; siRNA: 100 nM), ^****^ *p* ≤ 0.0001, one‐way ANOVA, Dunnett's multiple comparisons test. All experiments described are n = 3.

Subsequently, we proceeded to examine the cytotoxicity of both FNP1, FNP2, and NFNP using various cell lines. Our analysis revealed that the incorporation of fluorine into the synthesis of fluorinated IONPs had no harmful effects on D425 medulloblastoma cells, the BBB endothelial cell line, hCMEC/D3; and HCM3 microglia cells, even when exposed to FNP1 and FNP2 concentrations of up to 200 µg mL^−1^. However, it is worth noting that some level of toxicity was observed for NFNP at 200 µg mL^−1^ (**Figure** **3**A). The generation of reactive oxygen species (ROS) is one of the most well‐established ways that nanoparticles can damage cells and initiate apoptosis.^[^ ^26^ ^]^ We hypothesized that the addition of fluorine reduces cytotoxicity through suppression of the ROS process. First, we began by performing a cytotoxicity evaluation on FNP1 (representative fluorine nanoparticle) and NFNP using hCMEC/D3 cells (Figure S14A, Supporting Information). Both FNP1 and NFNP proved to be non‐toxic to cells up to 200 µg mL^−1^. To investigate the potential of fluorination in mitigating ROS activation, we exposed microglia (HCM3) and hCMEC/D3 cells to NFNP and representative fluorinated nanoparticle FNP1 and assessed the expression of CellROX Orange after a 2 h incubation period using confocal microscopy. The results demonstrate significant increases in ROS production at 40, 80, 120, 160, and 200 µg mL^−1^ Fe concentration, in the cells transfected with NFNP relative to control cells (*p* ≤ 0.05, *p* ≤ 0.0001, *p* ≤ 0.0001, *p* ≤ 0.0001, *p* ≤ 0.0001), whereas no change was observed in the cells transfected with FNP1 (Figure S14B, Supporting Information). This suggests a potential role for fluorine in “bio‐proofing” potential IONPs for siRNA delivery. Based on the cell viability results (Figure 3A), we then measured the uptake of nanoparticles without siRNA at 200 µg mL^−1^ for FNP1 and FNP2 and 150 µg mL^−1^ for NFNP in both D425 and HCM3 cells using ICP to analyze iron concentration (Figure 3B). All three types of IONPs showed cellular uptake in both cell lines. Whilst uptake was marginally greater for the NFNP, the results were not significant.

To investigate whether the addition of fluorine during synthesis would facilitate more robust cellular uptake of siRNA, we transfected semi‐adherent D425 and adherent HCM3 cells with FNP1, FNP2, and NFNP complexed to Alexa Fluor 647 (AF647) labeled siRNA (AF647 siRNA, red) and used confocal microscopy to observe uptake (Figure 3C). When FNP1‐siRNA complexes were exposed to cells, high levels of intracellular fluorescence were detected with a distinct punctate pattern, normally associated with endosomal entry.^[^ ^25^ ^]^ Whereas FNP2‐siRNA complexes instead displayed a more localized profuse pattern that suggested aggregation at the membrane (green), No uptake was detected for the NFNP‐siRNA, suggesting that even though NFNP was internalized by cells, it was ineffective at delivering the siRNA into the cells. Using live imaging we visualized the internalization of siRNA in cells treated with FNP1‐siRNA and membrane aggregation with sparse uptake for FNP2‐siRNA and NFNP‐siRNA, respectively (Movie S1‐S3, Supporting Information). Observations for FNP1, FNP2, and NFNP were consistent for D425 and HCM3 cell types. To see if this result was also true for other cancer cells, we also tested uptake for FNP1 complexed to AF647 siRNA in MCF7 breast cancer and HCT116 colon cancer cells using the same concentrations and transfection protocols. The results obtained were equivalent to the results obtained for D425 and HCM3, respectively – demonstrating the utility of FNP1 as a platform for delivering siRNA to different cancer cell populations (Figure S15, Supporting Information).

To confirm and further quantify our observations from the confocal microscopy analysis of medulloblastoma cells, we used flow cytometry to measure the uptake of FNP1, FNP2, and NFNP complexed to AF647 siRNA in D425 cells. As expected, FNP1‐siRNA complexes performed best, with ≈100% of cells emitting fluorescence (4 h). Whereas both FNP2 and NFNP complexes performed poorly, averaging ≈30% and ≈10%, respectively – for cells positive for the presence of siRNA (4 h) (*p* ≤ 0.0001) (Figure 3D). At 24 h none of the cells in the FNP2 and NFNP groups were positive for AF647 siRNA. Notably, the number of cells in the FNP1 group positive for fluorescence at 24 h was lower than for 4 h. Additionally, the median fluorescence intensity had fallen significantly (*p* ≤ 0.0001) (Figure 3E). These findings led us to hypothesize that some of the FNP1‐siRNA complexes had been exocytosed from the D425 cells 24 h post‐transfection.

To examine whether we could observe the siRNA leaving the cells, we first administered lysotracker dye to HCM3 cells following transfection with FNP1‐siRNA complexes, examining the cells using confocal microscopy at 24 h (Figure 3F). HCM3 cells were used in this experiment because they are adherent cells with a large cytoplasmic area making them optimally conditioned to determine the appearance of lysosomes. We observed colocalization between lysosomes (green) and siRNA (red) in four regions of interest which, when measured using Image J and the JACP plugin resulted in a colocalization measurement of ≈0.55 (Pearson's coefficient) (Figure 3F). Next, to quantitatively examine this effect in BBB cells, we transfected hCMEC/D3 cells with FNP1‐AF647 siRNA using immunofluorescence to determine exocytosis. Following 4 h of incubation, the transfection reagent was removed and replaced with fresh media. The fresh media was subsequently collected 2, 4, and 24 h post‐transfection. At the 2 h time point, 7.4 nM of siRNA was present in the fresh media. At 4 h 13.1 nM, and 24 h, 15.5 nM of siRNA was identified in the media – suggestive of a steady rate of exocytosis over time (Figure 3G). These results, therefore, support our hypothesis that the FNP1‐siRNA complexes are being exocytosed from cells over a 24 h period, a process necessary for siRNA‐based nanomedicines tuned toward crossing the BBB.

### 2.6 FNP1‐siRNA Complexes Successfully Traverse an In Vitro BBB and are Further Taken Up by Medulloblastoma Cells

**Figure 4:**
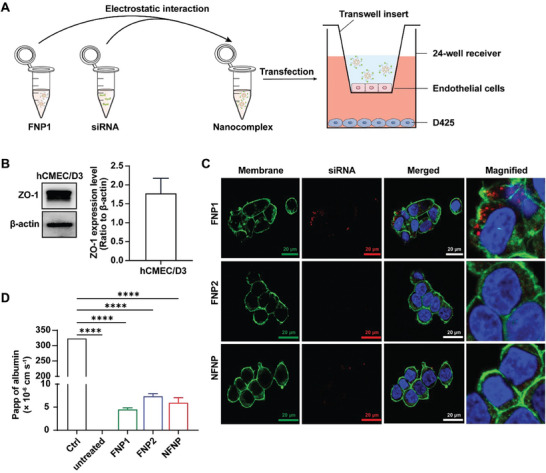
Fluorinated nanoparticles cross an intact in vitro BBB and internalize into medulloblastoma cells once across. A) Schematic diagram depicting the overall experimental design. B) ZO‐1 expression as a marker for tight junction formation. C) Representative confocal microscopy image demonstrating uptake of siRNA (red) when siRNA is delivered by FNP1. No visible uptake in cells exposed to FNP2 and NFNP, respectively (scale bar: 20 µm). D) Graph demonstrating apparent permeability as a measure of BBB integrity after transfection of BBB, ^****^ *p* ≤ 0.0001, one‐way ANOVA, Tukey's multiple comparisons test. All experiments described are n = 3.

Nanoparticle‐siRNA therapies tuned toward brain cancer treatment must be able to cross the BBB by facilitating endocytosis and exocytosis of the BBB endothelial cells. The complexes need to remain intact during this process. The ability to endocytose and exocytose the whole complex prior to entering cancer cells presents as one of the greatest challenges and thus, one of the most important early parameters to be explored. Importantly, our current results would indicate that FNP1‐siRNA complexes display the necessary characteristics for transcytosis of BBB microvasculature. Having established uptake and exocytosis of AF647 siRNA in the representative brain microvascular hCMEC/D3 cells (Figure S15, Supporting Information; Figure 3G), we next investigated whether the FNP1‐siRNA complexes were able to pass through a fully functional BBB. For this, we developed an hCMEC/D3‐derived in vitro model using a transwell insert (**Figure** **4**A). After examining the functional presence of tight junction proteins (ZO‐1) by western blotting (Figure 4B), we investigated the ability of FNP1, FNP2, and NFNP complexed to AF647 siRNA to cross through the in vitro model and enter medulloblastoma cells below. FNP1‐siRNA complexes were the only nanoparticles that showed visible fluorescence in the D425 medulloblastoma cells (Figure 4C). Importantly, fluorescence appeared to be localized to the cytoplasm suggesting that FNP1 was able to cross an intact BBB and subsequently deliver siRNA to brain cancer cells. To ensure that the observed effects were the result of transcytosis and not a result of the BBB becoming compromised by the transfection, we performed a permeability assay at 24 h. The results confirmed that the BBB remained intact throughout the experimental period (Figure 4D).

### 2.7 Fluoropolymer Engineered IONPs Deliver Functionally Active siRNA at pH 6.7

**Figure 5:**
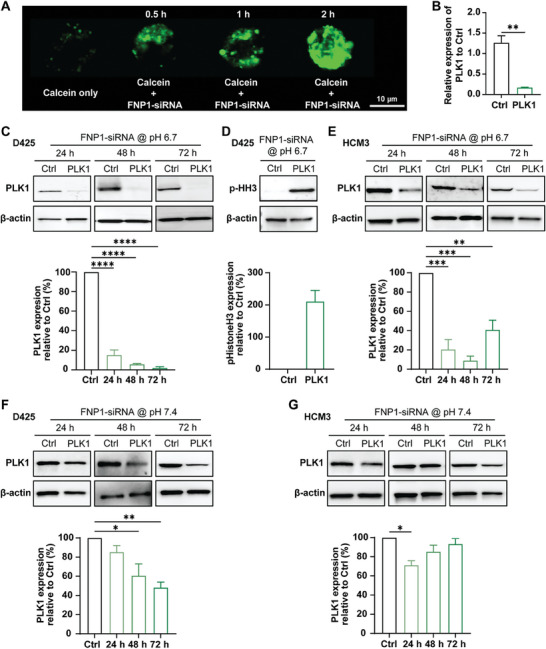
Extracellular pH 6.7 facilitates potent translational block following transfection with FNP1‐siRNA. A) Representative confocal microscopy image showing insurgence of calcein‐based fluorescence signal (green), demonstrating effective endosomal rupture in a D425 cell at 2 h post‐transfection (FNP1: 200 µg mL^−1^, siRNA: 100 nM) at pH 6.7 (scale bar: 10 µm). B) *Plk1* gene expression 24 h post‐transfection with FNP1‐PLK1 siRNA (200 µg mL^−1^; 100 nM) relative to FNP1 delivering control (Ctrl) siRNA at pH 6.7 (^**^ *p* ≤ 0.01). C) Representative western blots and corresponding densitometry graphs. D425 cells transfected at pH 6.7 demonstrating a significant 85% block in protein translation (^****^ *p* ≤ 0.0001) at 24 h, and 94% and 98% block in protein translation at 48, and 72 h respectively (^****^ *p* ≤ 0.0001). D) Representative western blot and densitometry graph of downstream PLK1 target, p‐HH3 at 24 h post transfection of D425 cells at pH 6.7. E) Representative western blots and corresponding densitometry graphs of HCM3 cells 24–72 h post‐transfection at pH 6.7 demonstrating a significant block in protein translation 24–48 h (^***^ *p* ≤ 0.001). However, by 72 h PLK1 protein expression begins to rise again (^**^ *p* ≤ 0.01). F) and G) Representative western blots and corresponding densitometry graphs showing PLK1 protein expression in F) D425 cells and G) HCM3 cells transfected with FNP1‐PLK1 siRNA nanoparticles at pH 7.4. D425 demonstrates a partial block in protein expression 24–72 h post‐transfection which is less in HCM3 cells (^*^ *p* ≤ 0.05, ^**^ *p* ≤ 0.01). Statistics used in these experiments are one‐way ANOVA and Dunnett's multiple comparisons test. All experiments described are n = 3.

Nanoparticles must be equipped to function well under acidic conditions within the tumor microenvironment – to first be endocytosed into cancer cells and then be able to be effectively released from the endosomes and deliver siRNA into the cytoplasm. To mimic the conditions of the tumor microenvironment we conducted experiments showing endosomal rupture and cytoplasmic release, and gene silencing experiments in OptiMem transfection media at ≈pH 6.7. Given the significant lack of uptake observed for both FNP2 and NFNP, endosomal rupture and gene silencing experiments were focused principally on FNP1‐siRNA complexes. To observe endosomal rupture occurring in medulloblastoma cells following uptake, D425 cells were transfected with FNP1‐AF647 siRNA in OptiMem ≈pH 6.7 containing 150 µg mL^−1^ Calcein dye (green). Cells were imaged live at 0.5, 1, and 2 h post‐transfection. The early stages of endosomal rupture could be witnessed at 0.5 h; however, the greatest observation of endosomal rupture was at 2 h post‐transfection (**Figure** **5**A).

To determine the ability of FNP1 to deliver therapeutically relevant siRNA we chose to deliver siRNA targeting the oncogene *Polo‐Like Kinase 1 (Plk1)*. PLK1 is one of the most well‐characterized cell cycle regulators in cancers including medulloblastoma, and numerous studies have identified that inhibition results in cancer‐specific cell death.^[^ ^27^ ^]^ For this experiment, D425 medulloblastoma cells were transfected with FNP1‐PLK1 siRNA. Gene silencing of *Plk1* measured at 24 h demonstrated a significant silencing effect at the gene level (*p* ≤ 0.01) when measured against FNP1 delivering non‐targeting control (Ctrl) siRNA (Figure 5B). Subsequently, when we used densitometry as a semi‐quantitative measure of protein expression after 24 h, PLK1 was reduced by ≈85% in D425 (*p* ≤ 0.0001) cells relative to the Ctrl siRNA group and by 48 h, protein translation was further reduced to ≈94% (*p* ≤ 0.0001). An almost complete translational block on protein synthesis for PLK1 was observed at 72 h (≈98%) (*p* ≤ 0.0001), suggesting that FNP1‐PLK1 siRNA was a highly potent therapeutic when complexes were administered at pH 6.7; an extracellular pH previously observed as the favored pH of cancer cells and associated with highly proliferative and metastatic cells (Figure 5C).^[^ ^28^ ^]^ Because of PLK1's fundamental role in cancer cell cycle progression, cell cycle arrest is a notable effect in cancer cells following inhibition or gene silencing.^[^ ^23^, ^29^ ^]^ Whilst specificity of our chosen PLK1 siRNA sequence has been previously reported, to confirm the specificity of the whole complexes toward the *Plk1* gene, mitotic arrest marker, phosphorylated Histone H3 (p‐HH3) protein expression was also measured by western blot.^[^ ^23^ ^]^ The results demonstrate an ≈80‐fold increase in expression of p‐HH3 expression at 24 h relative to cells transfected with Ctrl siRNA, indicating that D425 cells had entered a phase of mitotic catastrophe, a known outcome in cancer cells following the absence of PLK1 (Figure 5D).^[^ ^30^ ^]^

We further examined the effect of FNP1‐PLK1 siRNA in microglia. Here too, a significant knockdown was observed at 24 h (*p* ≤ 0.001). However, unlike the medulloblastoma cells, knockdown peaked at 48 h and then began to rise again at 72 h (Figure 5E). Phenotypically, whilst D425 cells entered apoptosis and numbers dwindled, microglia cell numbers remained consistent with negligible observed apoptotic events (data not shown). This outcome is in line with various studies that have examined the difference following inhibition of PLK1 in cancer cells and non‐cancer cells.^[^ ^27^, ^31^ ^]^ To confirm that FNP1 was primed specifically for silencing gene expression in conditions mimicking the tumor microenvironment rather than at pH 7.4 – as occurs in blood and healthy tissues, we measured the translational effect of gene silencing for *Plk1* when D425 and HCM3 cells were transfected in OptiMem pH 7.4 (Figure 5F,G). The block in protein expression was greatly reduced in both cell lines relative to that of the pH 6.7 data (Figure 5C,E).

### 2.8 Fluorination Improves Blood Circulation of IONPs

**Figure 6:**
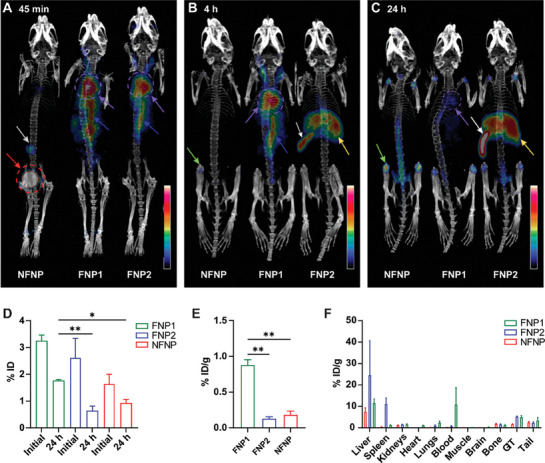
FNP1 shows lengthy in vivo circulation of ^89^Zr radiolabeled nanoparticles and accumulation in the brain A) 45 minutes, B) 4 h, and C) 24 h post‐injection. Color scale bar represents the intensity of signal proportional (% ID g^−1^). Images are scaled to upper limit (white) 35% and above, 10% (black). Blood pool activity has been removed by bringing the limit from 0% to 10%. Red arrows and dotted circles demonstrate rapid clearance of NFNP to the bladder. The white arrows demonstrate splenic uptake. Purple arrows and dotted circles outline uptake seen in the heart and abdominal aorta (blue arrow). Bone (green arrows) is highlighted on 4 B) and 24 h C) images of the NFNP. Liver (yellow arrow). Grey arrow represents an image artifact. D) In vivo PET radiolabeled NP within the brain at initial and 24 h post‐injection for FNP1 (n = 3), FNP2 (n = 2), and NFNP (n = 2) injected mice. Retention of radiolabeled FNP1 within the brain of injected mice exhibits significant retention to FNP2 (^**^ *p* ≤ 0.01) and to NFNP (^*^ *p* ≤ 0.05). FNP2 and NFNP demonstrate no significant difference in retention, calculated and expressed as a percentage of the initial injected dose (% ID), one‐way ANOVA, and Tukey's multiple comparisons test. E) Ex vivo retention of radiolabeled NP within the brain of FNP1 injected mice exhibit significant retention to both FNP2 and NFNP (^**^ *p* ≤ 0.01). FNP2 and NFNP demonstrate no significant difference in retention, calculated and expressed as a percentage of the initial injected dose per gram (% ID g^−1^), one‐way ANOVA, and Tukey's multiple comparisons test. F) Ex vivo samples were weighed, and decay was corrected to calculate % ID g^−1^ for the organ/tissue sample; Liver, Spleen, Kidneys, Heart, Lungs, blood, Muscle (thigh), Brain, Bone (femur), Gastrointestinal tract (GIT), and Tail.

Chelator‐free radiolabeling of ^89^Zr polymeric fluorinated and non‐fluorinated nanoparticles was performed. A high labeling efficiency was achieved through the Ligand Anchoring Group Mediated Radiolabeling (LAGMERAL) method.^[^ ^32^ ^]^ A resulting radiochemical purity of >97% was obtained on radio‐TLC prior to in vivo administration (Table S4 and Figure S16, Supporting Information). To visualize the behavior and pharmacokinetics of FNP1, FNP2, and NFNP in pre‐clinical murine models, PET/CT imaging was performed longitudinally. Volumetric regions of interest (ROI) were drawn over the organs of interest; heart, liver, spleen, kidney, bladder, and lung on images acquired over the initial 45 min (dynamic), then 4 and 24 h post administration (**Figure** **6**A–C). Activity concentrations were calculated and expressed as a percentage of the injected dose, per gram of body weight (%ID g^−1^) (Figure S17, Supporting Information).

^89^Zr‐NFNP demonstrated a significant increase in blood pool activity throughout the systemic circulation and clearance via the kidneys on the initial 45 min dynamic imaging. Increased ^89^Zr FNP2 signal was observed in bone joints throughout the skeleton over time, which was further confirmed from tissues sampled for ex vivo analysis. This pattern of biodistribution demonstrated minimal systemic circulation at 4 and 24 h post‐injection, possibly due to the nature of the non‐fluorinated polymer. In contrast, ^89^Zr‐ FNP1 had a consistent systemic distribution over the 24 h period. An increased PET signal was observed in the heart and large blood vessels in early dynamic images and 4 h images, suggesting a longer residence time in the blood pool. Images acquired at 24 h post‐injection revealed minimal liver or splenic retention of the tracer, suggesting potential uptake by the mononuclear phagocytic system. Similarly, initial dynamic imaging of ^89^Zr‐FNP2 illustrated elevated systemic circulation relative to the NFNP, evidenced by marked intensity in the abdominal aorta and heart with no evidence of renal excretion. However, images acquired 24 h post‐injection revealed low systemic circulation with no heart or large vessel accumulation seen. This was accompanied by increased liver and spleen uptake (>35% ID g^−1^). Blood half‐life (*t*_1/2_) was calculated from the heart time activity curve (TAC) demonstrating FNP1: 250 min, FNP2: 80 min, and NFNP: 50 min, respectively. Collectively, the results for FNP1 suggest excellent biodistribution and a superior systemic circulation relative to FNP2 and NFNP and strongly support FNP1 as a nanoparticle able to extend the opportunity for siRNA delivery in vivo.

### 2.9 Fluoropolymer Engineered IONPs Cross the BBB in Healthy Mice

To determine whether FNP1 was physiochemically equipped to cross an intact BBB in vivo, PET imaging post‐processing quantification was performed 45 min and 24 h post‐injection (% ID). ROI volumes were calculated within the brain tissue avoiding bone and cerebral blood circulation, and were decay‐corrected for delayed imaging. FNP1 presented the highest percentage of nanoparticles in the brain after the initial injection (3.24% ID). Furthermore, 1.84% ID of FNP1 remained within the brain a the 24 h time point. Meanwhile, FNP2 and NFNP show similar lower‐level uptake and elimination over the 24 h period (<1% ID) (Figure 6D). Similarly, ex vivo biodistribution analysis of the healthy brain tissue demonstrated comparable results to the in vivo data, where FNP1 injected mice exhibit significant retention (*p* ≤ 0.01) of radiolabeled nanoparticles at 24 h post‐injection with a mean retention of 0.88% of the initial injected dose, in comparison to FNP2 and NFNP injected mice (0.13% and 0.19%, respectively) (Figure 6E).

Multiorgan/tissue biodistribution analysis was performed using a gamma counter. %ID g^−1^ plotted for graphical representation in Figure 6F was comparable with PET imaging results in Figure 6C. The blood collection analysis showed a 7.76‐fold increase of radiolabeled nanoparticles in systemic circulation for FNP1 when compared to NFNP and an 18.33‐fold increase when compared to FNP2.

### 2.10 Fluoropolymer Engineered IONPs for In Vivo MR Imaging and Gene Therapy of Medulloblastoma

**Figure 7:**
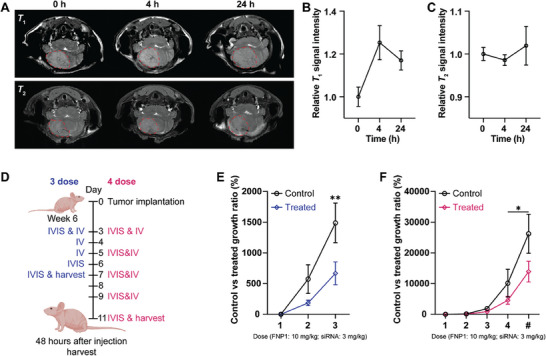
FNP1 can be used as an MRI probe and delivery vehicle for therapeutic siRNA in an orthotopic medulloblastoma mouse model. A) *T*_1_‐ and *T*_2_‐weighted MR images of a tumorous brain section of mice bearing medulloblastoma acquired before and at different time points after intravenous injection of 10 mg kg^−1^ FNP1. Red dotted line represents tumor area. B) Graph showing relative *T*_1_ signal intensity at time points 0, 4, 24 h. C) Graph showing relative *T*_2_ signal intensity at time points 0, 4, and 24 h. D) Schematic illustrating two different dosing regimens. E) Graphed data showing significantly slower growth in mice treated with FNP1‐ PLK1 siRNA over 3 days compared to control. For the x‐axis, 1: IVIS before treatment; 2: 24 h after 3 dose treatments; and 3: 48 h after 3 doses treatments, ^**^ *p* ≤ 0.01, two‐way mixed ANOVA with uncorrected Fisher's LSD test. F) Graphed data showing significantly slower growth in mice treated with four doses of FNP1‐ PLK1 siRNA over 8 days compared to control. For the x‐axis, 1: IVIS before treatment; 2: 48 h after 1 dose treatments; 3: 48 h after 2 doses treatments; 4: 48 h after 3 doses treatments; #: 48 h after 4 doses treatments, ^*^ *p* ≤ 0.05, two‐ way mixed ANOVA with uncorrected Fisher's LSD test. All experiments described are n = 3 unless otherwise indicated.

We next sought to determine whether FNP1 could take advantage of the significant circulatory times that we observed in the PET‐CT study and be successfully implemented as a negative contrast imaging probe as well as a therapeutic siRNA delivery vehicle. For the MRI investigations, mice with orthotopic medulloblastoma tumors (n = 3) were administered FNP1 (10 mg kg^−1^) via tail intravenous injection, and the *T*_1_‐ and *T*_2_‐weighted MR image acquisition was performed at different times points after injection (**Figure** **7**A). According to the *T*_1_‐weighted MR images and temporal relative *T*_1_ signal intensity illustrated in Figure 7A,B, FNP1 enhanced the *T*_1_ signal intensity at the tumor site 4 h post‐injection. The signal intensity subsequently decreased over time, indicating the accumulation and clearance of the FNP1.^[^ ^33^ ^]^ Additionally, as shown in Figure 7A,C, the *T*_2_ signal decreased at 4 h post‐injection of FNP1 and increased over time.^[^ ^33b^ ^]^ Furthermore, the changes of *T*_1_, *T*_2_ values support that FNP1 has greater *T*_2_ contrast properties. A *T*_1_ map was used to establish *T*_1_ values; however, no statistical significance was determined (Figure S18A, Supporting Information). While at the 4 h time point, mice demonstrated a significant fall in *T*_2_ values (*p* ≤ 0.05) at the tumor site (Figure S18B, Supporting Information). Twenty‐four‐hour images demonstrated a shorting in *T*_2,_ that was slightly greater after administered FNP1 (*p* ≤ 0.05), indicating the presence of FNP1 in tumor tissue.^[^ ^34^ ^]^

To show that FNP1 was able to deliver therapeutically relevant siRNA in vivo, mice with orthotopic tumors were administered FNP1 delivering PLK1 siRNA, previously established to pressure cancer cells into mitotic catastrophe and slow growth in aggressive cancers.^[^ ^25^ ^]^ Two treatment regimens were investigated (Figure 7D). The first, three intravenously (IV) delivered FNP1‐siRNA over three consecutive days. The second, four IVs delivered FNP1‐siRNA every other day for eight days. Using luciferase expression as a marker for tumor burden, we observed that whilst mice administered saline (n = 3) saw a rapid increase in tumor growth – in line with previously reported data,^[^ ^35^ ^]^ mice administered with FNP1‐siRNA over three consecutive days (n = 4) demonstrated a significantly slower rate of growth (^**^ *p* ≤ 0.01) (Figure 7E). Moreover, this effect was also observed when n = 5 mice were treated with FNP1‐siRNA in the second regimen of four treatments over eight days (^*^ *p* ≤ 0.05) (Figure 7F). Lastly, to examine whether damage had occurred to the architecture of the brain and major organs (heart, lung, liver, kidney, and spleen), as a consequence of the eight‐day exposure, we also performed H&E staining of the organs (Figure S19, Supporting Information). No gross changes to the structure of each organ were observed, suggesting good early time point biocompatibility.

## 3 Discussion

Whilst siRNA‐based therapeutics have reached their potential in treating various hepatocellular‐initiated maladies, it has remained a challenge to achieve the same level of success for siRNA therapeutics aimed at brain cancers.^[^ ^36^ ^]^ This is because although siRNA‐based gene therapy holds significant promise for treating brain cancers, including medulloblastoma, it faces unique challenges on its journey toward clinical application. Foremost among these challenges is the necessity to breach the BBB prior to effective delivery toward the diseased site within the brain. Nanoparticles are one of the best ways to overcome this challenge; however, there remains a lack of biologically tuned nanoparticle vectors that are stable and protective of siRNA in the blood, able to cross the BBB and tumor vasculature; and are optimally primed for delivery of siRNA in more acidic conditions associated with the tumor microenvironment.

Fluoropolymers have garnered significant interest due to their impressive serum stability, arising from their bio‐inert and anti‐fouling characteristics. Prior research has demonstrated that fluoroalkyl ligands can enhance the transfection efficiency of cationic polymers by promoting complex stability, facilitating increased cell membrane penetration, and aiding in endosomal escape.^[^ ^37^ ^]^ Among various fluoropolymers, PFPE polymers have previously been employed as drug delivery carriers and contrast agents in ^19^F MRI, showcasing substantial promise for the in vivo delivery of payloads.^[^ ^38^ ^]^ Based on these rationales, we proposed the development of a multifunctional fluoropolymer combining the cationic and pH‐responsive components for siRNA binding and controlled release, OEGA for water solubility and biocompatibility, as well as PFPE for serum stability and cellular uptake. This polymer was then grafted onto the surface of a core particle, IONP, to create a distinctive surface structure. This structure allows the hydrophobic PFPE and hydrophilic monomers to be oriented toward the outer layer, maximizing their potential for anti‐biofouling, membrane penetration, and endosome escape within the cytoplasm. Here we show that the specialization and methodology employed to develop our nanoparticles has resulted in the development of a robust delivery vector capable of crossing the BBB in vivo and subsequently working as an MRI probe. Moreover, performing the function of a delivery vehicle for therapeutically active siRNA.

A hallmark of the tumor microenvironment is a reversed pH gradient whereby cancer cells reside in a lower extracellular pH of ≈6.7–7.1 and display a higher intracellular pH of 7.4.^[^ ^28c^ ^]^ Normal cells, such as those of the BBB on the other hand exhibit a higher extracellular pH of 7.4 and a lower intracellular pH of 7.2.^[^ ^39^ ^]^ Therefore, we first determined the most affective delivery platform for crossing the BBB at pH 7.4. Next, we examined the potential of our nanoparticles to elicit a potent gene‐silencing effect at low extracellular pH conditions that aim to mimic the tumor microenvironment (pH 6.7). Our investigations revealed that the fluoropolymers grafted IONPs facilitate transportation across the BBB by enhancing the cellular uptake of IONPs‐siRNA assemblies in vitro. Notably, at pH 7.4 the assemblies were exocytosed with little gene silencing observed. This was because the introduction of the cationic polymer component, alongside the fluoropolymer introduced a pH‐responsive mechanism for a more targeted siRNA release profile tuned toward the tumor microenvironment. Unlike at pH 7.4, when IONP‐siRNA complexes were used to transfect tumor cells at pH 6.7 they exhibited a remarkable capacity to induce gene silencing and a block in protein translation in medulloblastoma cells.

During the in vivo PET biodistribution study, we first observed negligible uptake within the lungs. Prompt accumulation within the lungs typically indicates poor colloidal stability or aggregation of the particles. Therefore, this finding allowed us to speculate that FNP1 nanoparticles do not cause significant aggregation and thus reduce the potential to cause nanoparticle‐induced lung inflammation – as previously reported for a range of other nanoparticle vectors.^[^ ^40^ ^]^ Also, degradation of the bidentate ^89^Zr─O bonds of the polymer coating was observed, however, this can be explained by the stronger osteophillic properties of the ^89^Zr^4+^ metal cation in vivo, resulting in skeletal remineralization in active bone and joints.^[^ ^41^ ^]^ Brant et al. explain the polymer complex, in the absence of an octadentate chelating agent, has an incomplete co‐ordination sphere of the radiometal, leading to unfavorable and unspecific bone uptake of the osteophillic radiometal and is not reflective of the nanoparticle behavior.^[^ ^42^ ^]^ Meanwhile, rapid renal clearance as demonstrated on ^89^Zr‐NFNP imaging, can legitimately be attributed to the smaller particle size, and as such, exhibited expected pharmacokinetic behavior.

The poor biodistribution results for FNP2 were similar to those previously observed by McDonagh et al. using PET‐CT. ^89^Zr labeled dextran‐10‐PEG coated nanoparticles, which they attributed to ^89^Zr rapidly detaching from the nanoparticles.^[^ ^43^ ^]^ However, in contrast to the McDonagh et al. study and FNP2, FNP1 demonstrated negligible immediate clearance activity, and importantly, minimal bone uptake suggestive of continued ^89^Zr attachment to FNP1 over time. Collectively, these data highlight the outstanding ability of FNP1 to remain within systemic circulation for a prolonged period‐evading immune clearance and enhancing siRNA delivery efficiency.

Whilst literature is limited regarding intravenously delivered PET radiolabeled nanoparticles crossing the BBB for orthotopic medulloblastoma models and healthy non‐ immunocompromised mice, our FNP1 nanoparticles were demonstrated to perform better than several alternative studies. For example, Hong group systemically injected functionalized Copper‐64 (^64^Cu) gadofullerene nanoparticles into a xenograft glioblastoma model. Their ex vivo data demonstrated brain tissue retention below 0.7% ID g^−1^,^[^ ^44^ ^]^ similar to FNP2 and NFNP. Comparable results to these in orthotopic xenograft glioblastoma tumor models were also reported by Pang et al., by injecting double the radioactive dose with respect to our study yielded below 0.14% ID g^−1^.^[^ ^45^ ^]^ Meanwhile, Seo et al. investigated the ability of targeted ^64^Cu radiolabeled adeno‐associated virus capsids aimed to enhance brain delivery via the LY6A receptor.^[^ ^46^ ^]^ The results of this study are comparable to FNP1 at 24 h (1.84%).

For MRI previous data suggests that *r*_1_ and *r*_2_ values could be affected by several factors such as size and frequency.^[^ ^47^ ^]^ In this study, the reported difference in *r*_1_, and *r*_2_ values observed between FNP1 and FNP2 can be explained by the specific structure of surface capping polymers associated with the translational diffusion time (τD) for water molecules to pass through the fluoropolymer coating.^[^ ^9j^ ^]^ Specifically, the formation of a hydrophobic outer‐sphere, which predominantly influences the proton relaxation of IONPs^[^ ^9j^ ^]^ is expected to enhance the *r*_2_ and *r*_1_ values in FNP2 due to the increased τD value. Whilst the ≈3 nm‐sized iron oxide utilized in this study will produce *T*_1_ signals under clinically used 3.0 T MRI instruments for animal studies in our paper, a 7.0 T MRI was employed, where the higher magnetic field induces a more pronounced *T*_2_ effect.^[^ ^48^ ^]^

In our final study, we investigated whether FNP1 was capable of delivering siRNA able to slow down the growth rate of aggressive, fast‐growing type 3 medulloblastoma. Using siRNA targeting established oncogene *Plk1*, we were able to show a significant deceleration in growth rate when compared to mice receiving saline. Taken together these results are indeed promising; however, before FNP1's potential can be fully realized, additional more translationally relevant experiments beyond the scope of this proof‐of‐concept study must be performed. For instance, while tumors were not completely eradicated, our findings do suggest that FNP1‐siRNA may have an important role to play as a sensitizing agent prior to the harsh onslaught of chemotherapeutics regimens. We hypothesize that by creating a destabilized tumor environment, chemotherapeutics may work more effectively. Testing this hypothesis should form the basis of future investigations. Additionally, it is our belief that immune tolerance should next be examined, and although our pharmacokinetic study and H&E staining of organs presented a short‐term look at the potential toxicity and overall behavior of our nanoparticles, long‐term effects of exposure should be investigated. This is especially important given that siRNA therapeutics may need to be administered repeatedly. The experiments described in this paragraph will form the basis of our ongoing studies to bring FNP1 as a delivery vehicle for siRNA toward the clinic. In summary, the successful proof of concept study results presented here support the premise of utilizing FNP1 for brain tumor‐specific siRNA gene silencing. Once translationally important experiments have been optimized and executed FNP1 could pave the way to effectively treating not just medulloblastoma patients more effectively, but all brain cancer patients.

## 4 Experimental Section

### Materials

All the chemicals and solvents were purchased from Sigma‐Aldrich or Merck and used as received unless otherwise stated. Hydroxy‐terminated perfluoropolyether (PFPE‐OH, PFPE AL‐2, *M*_w_ ≈ 2000 g mol^−1^, CAS Number: 126066‐30‐6) was purchased from The Chemours Company.

### Methods: Synthesis of 2‐(((Butylthio)carbonothioyl)thio)Propanoic Acid (BTPA) and BTPA‐PFPE as Chain Transfer Agent (CTA)

BTPA and BTPA‐PFPE RAFT agents were synthesized as pervious described.^[^ ^9^, ^49^ ^]^ Briefly, PFPE‐OH (10 g, 5 mmol), BTPA (2.38 g, 10 mmol), and DMAP (183.26 mg, 1.5 mmol) were dissolved in α,α,α‐trifluorotoluene (TFT) (60 mL) in a 250 mL round bottom flask and pre‐cooled at 0 °C with stirring. *N*‐(3‐(dimethylamino)propyl)‐*N*’ethylcarbodiimide hydrochloride (1.917 g, 10 mmol) dissolved in anhydrous dichloromethane (DCM) (20 mL) was added dropwise into the reaction mixture. The reaction was performed at room temperature for 48 h. The BTPA‐PFPE RAFT agent was obtained by precipitation into a large amount of methanol five times, followed by drying overnight using an oil pump at room temperature.

### Methods: Synthesis of 2‐(Dimethoxyphosphoryl)Ethyl Acrylate

The synthesis of 2‐(Dimethoxyphosphoryl)ethyl acrylate was described previously.^[^ ^9b^ ^]^ Briefly, acrylic acid (2.80 g, 38.93 mmol) and EDC (6.05 g, 38.93 mmol) were mixed in tetrahydrofuran (THF) (20 mL) under an ice bath. DMAP (0.20 g, 1.62 mmol, 0.05 equiv. to dimethyl (2‐hydroxyethyl)phosphonate) was added to the reaction mixture as a catalyst. After 2 h, dimethyl (2‐hydroxyethyl)phosphonate (5.00 g, 32.45 mmol) dissolved in THF (20 mL) was added dropwise, and the reaction was left at room temperature for 24 h after complete addition. The THF was removed by evaporation and re‐dissolved in DCM (30 mL). The crude mixture was obtained after washing with saline solution (3 × 40 mL) and drying over anhydrous MgSO_4_. The pure product was obtained by FLASH column chromatography (10% to 40% DCM/THF) as a colorless liquid. ^1^H NMR (400 MHz, CDCl_3_): δ = 6.43 (1H, dd, *J* = 17.4, 1.3 Hz, vinyl), 6.12 (1H, dd, *J* = 17.3, 10.4 Hz, vinyl), 5.86 (1H, dd, *J* = 10.4, 1.36 Hz, vinyl), 4.40 (2H, dt, *J* = 13.1, 5.8 Hz, COOCH_2_), 3.77 (6H, d, *J* = 11.0 Hz POCH_3_), 2.22 (2H, dt, *J* = 19.0, 7.5 Hz, CH_2_CH_2_P) ppm.

#### Methods: Synthesis of Block Polymers using RAFT Polymerization

A typical RAFT polymerization was conducted to synthesis the multifunctional block polymers. Briefly, BTPA‐PFPE RAFT agent (400 mg, 0.18 mmol), AIBN (5.97 mg, 0.036 mmol), poly(ethylene glycol) methyl ether acrylate (OEGA, *M*_n_ 480) (1047.3 mg, 2.18 mmol), and 2‐(dimethylamino)ethyl acrylate (DMAEA) (156.2 mg, 1.08 mmol) were mixed in TFT (1.5 mL) and *N, N*‐Dimethylformamide (DMF) (0.5 mL) in a 25 mL round bottom flask. The solution was then sealed and pre‐cooled under an ice bath, followed by deoxygenating for 15 min with argon. The reaction solution was then transferred to 70 °C oil bath and left for 4 h. The final product PFPE‐Poly(OEGA_12_‐*co*‐DMAEA_6_) was purified by precipitation in a mixture of diethyl ether and n‐hexane (1:1) three times and dried in vacuo.

The PFPE‐Poly(PA_6_) and Poly(OEGA_11_‐*co*‐DMAEA_5_) were also polymerized using BTPA‐PFPE and BTPA as RAFT agents, respectively. And the second polymerization was also conducted following a similar procedure to obtain the final products, PFPE‐Poly(PA_6_‐*b*‐(OEGA_12_‐*co*‐DMAEA_6_)), PFPE‐Poly((OEGA_11_‐*co*‐DMAEA_6_)‐*b*‐PA_5_), and Poly((OEGA_11_‐*co*‐DMAEA_5_)‐*b*‐PA_8_), respectively.

#### Methods: Deprotection of Block Polymers

Typically, PFPE‐Poly(PA_6_‐*b*‐(OEGA_12_‐*co*‐DMAEA_6_)) (1.00 g, 0.10 mmol) was dissolved in DCM (5 mL) under an ice bath. The reaction solution was sealed with septa, followed by degassing with argon for 10 min. Then, bromotrimethylsilane (TMSBr) (364.68 mg, 2.4 mmol, 4 equiv. to phosphonate groups) was dissolved in DCM (2 mL) and added dropwise to the reaction mixture. After 24 h reaction at room temperature, DCM was removed by evaporation, and methanol (5 mL) was added for another 4 h. The final polymer was obtained by precipitation in a mixture of diethyl ether and n‐hexane (1:1) and drying in vacuo.

#### Methods: Preparation of IONPs by Thermal Decomposition

A 3 nm IONPs were synthesized according to the previous report.^[^ ^17a^ ^]^ The procedure is further optimized as follows: iron‐oleate complex (1.8 g, 2 mmol), and oleyl alcohol (3.22 g, 12 mmol) were dissolved in diphenyl ether (20 mL). Then the reaction mixture was heated up to 90 °C and degassed by nitrogen for 2 h. After degassing, the mixture was further heated to 250 °C with a constant heating rate of 20 °C per min and kept at that temperature for 30 min under the inert atmosphere. The final mixture was cooled to ambient temperature, and acetone (50 mL) was added to precipitate the 3 nm IONPs. The 3 nm IONP precipitation was re‐suspended in THF after centrifugation and washed three times with acetone. The iron concentration was determined by Thermo Scientific iCAP PRO inductively coupled plasma‐optical emission spectrometry (ICP‐OES).

#### Methods: Ligand Exchange to Engraft Polymers onto IONPs

A 3 nm IONPs (1 mg) in chloroform (CHCl_3_) (1 mL) was added to deprotected polymer (10 mg) dissolved in CHCl_3_ (2 mL). The ligand exchange reaction was performed in a 50 °C oil bath for 24 h. The reaction product was precipitated in diethyl ether and n‐hexane (v/v 1:1), and centrifuged at 4000 rpm for 10 min. The final precipitation was re‐suspended by Milli‐Q water (5 mL). To discard any insoluble fragments, the product was centrifuged at 10 000 rpm. The supernatant was collected and purified by ultrafiltration with an Amicon Ultra centrifugal filter (MWCO, 100 kD) and centrifugation at 4000 rpm for 10 min, which was performed thrice to remove the excess free polymers. The concentration of purified product was determined by ICP‐OES.

#### Methods: Size and Zeta Potential before and after Complexation with siRNA

The size, polydispersity (PDI), and zeta potential of the nanoparticles were investigated before and after complexation with siRNA using Malvern ZS Zetasizer (Malvern Instruments Ltd.) at room temperature (25 °C). All measurements were collected in triplicate.

#### Methods: Transmission Electron Microscopy

Transmission electron microscopy (TEM) was used to observe the morphology of the nanoparticles. TEM images were obtained by a Hitachi HT7700 equipped with a tungsten filament as previously described.^[^ ^19^ ^]^

#### Methods: Gel Shift and siRNA Displacement Assays

The binding potential for siRNA was measured by gel shift assay as previously described.^[^ ^50^ ^]^ Briefly, different concentrations of nanoparticles based on iron concentration (0.3, 0.6, 1.3, 2.5, 5.0, and 10.0 µg mL^−1^) were complexed to siRNA (1 µm) for 20 min before being loaded onto a 2% (w/v) agarose gel for electrophoresis run at 20 V for 30 min.

A heparin displacement assay was conducted to determine the binding affinity between the nanoparticles and siRNA. Nanoparticles (50 µg) were complexed to siRNA (25 nM) for 20 min. Following complexation, heparin solution (0, 1, 2, 4, 8, 16, and 20 µg mL^−1^) was added to the nanoparticle‐siRNA complexes, and the samples were incubated for a further 30 min at 37 °C. A Ribogreen assay was used to examine displacement as per the manufacturer's instructions (Thermo Fisher Scientific Inc).

#### Methods: MRI Relaxivities of IONPs

The MRI relaxation properties were measured with a 300 mm‐bore 7.0 T MR scanner (Burker ClinScan, Germany) using an 86 mm coil. Different concentrations of FNP1, FNP2, and NFNP solutions were loaded in 5 mm NMR tubes, which were then parafilm and packed inside a 50 mL vial, and both spin‐lattice (*T*_1_) and spin‐spin (*T*_2_) relaxation times were acquired. The RARE sequence was used to obtain *T*_1_, and the measuring parameters were as follows: field of view (FOV) = 45 × 45 mm, slice thickness (SI) = 2 mm, repetition time (TR) = 200, 400, 800, 2000, and 5500 ms, echo time (TE) = 8.5 ms, flip angle = 180°. *T*_2_ relaxation time was assessed with the same sequence and the following parameters were applied: FOV = 45 × 45 mm, SI = 2 mm, TR = 2000 ms, TE = 8.5 – 93.5 ms, flip angle = 180°. The longitudinal and transverse relaxivities (denoted *r*_1_ and *r*_2_, respectively) were determined by *r_i_* = (1/*T*_i_–1/*T*_i0_)/*c*, in which *c* is the iron concentration of the IONPs in mm, *T*_i_ is the measured relaxation time at concentration *c*, *T*_i0_ is the relaxation time of water protons. The data were analyzed with Paravision 360 v3.4 (Bruker, Germany).

The same concentrations of nanoparticles were also measured with a 300 mm‐bore 9.4 T MR scanner (Burker Biospec, Germany) using an 86 mm coil. The RARE‐VTR sequence was used to obtain *T*_1_, and the measuring parameters were as follows: FOV = 45 × 45 mm, SI = 2 mm, TR = 20, 40, 80, 120, 240, 500, 1000, and 2000 ms, TE = 10 ms, flip angle = 180°. *T*_2_ relaxation time was assessed with a multi‐slice multi‐echo (MSME) sequence and the following parameters were applied: FOV = 45 × 45 mm, SI = 2 mm, TR = 2500 ms, TE = 10 – 800 ms, flip angle = 180°. The *r*_1_ and *r*_2_ were determined by *r_i_* = (1/T_i_–1/T_i0_)/*c*, in which *c* is the iron concentration of IONP in mm, *T*_i_ is the measured relaxation time at concentration *c*, *T*_i0_ is the relaxation time of water protons. The data were analyzed with Paravision 7 imaging software (Bruker, Germany).

#### Methods: Cell Culture

Medulloblastoma cells (D425) and brain endothelial cells (hCMEC/D3) were purchased from Merck (Australia). Microglia (HCM3), breast cancer cells (MCF7), colorectal cancer cells (HCT‐116), and human umbilical vein endothelial cells (HUVEC) were purchased from ATCC (USA). Dulbecco's Modified Eagle Medium/Nutrient Mixture F‐12 (DMEM/F‐12), Dulbecco's Modified Eagle's Medium (DMEM), Minimum Essential Medium (MEM) culture medium, and Fetal Bovine Serum (FBS) were supplied by Gibco (Grand Island, NY, USA). EndoGRO‐LS Complete Media Kit and Collagen Type I, Rat Tail were purchased from Merck (Australia).

D425 cells are cultured in DMEM/F‐12 supplemented with 10% of FBS. HCM3 cells and HCT‐116 cells were cultured in DMEM supplemented with 10% of FBS. MCF7 cells were cultured in MEM supplemented with 10% of FBS. For hCMEC/D3 cells and HUVEC cells culture, culture flasks were first coated with collagen type I in PBS (1:20, v/v) for a minimum of an hour at 37 °C. hCMEC/D3 cells and HUVEC cells were cultured in EndoGRO Basal medium (EBM) supplemented with FBS (2%), EndoGRO‐L supplement (0.2%), rh EGF (5 ng mL^−1^), ascorbic acid (50 µg mL^−1^), L‐glutamine (10 mm), hydrocortisone hemisuccinate (1 µg mL^−1^) and heparin sulfate (0.75 U mL^−1^) from EndoGRO‐LS Complete Media Kit. All cell lines were cultured in a humidified atmosphere of 5% CO_2_ at 37 °C.

#### Methods: Nanoparticle Cytotoxicity and Nanoparticle Uptake

The cytotoxicity of the nanoparticles was examined by AlamarBlue assay. Briefly, hCMEC/D3, HCM3, and D425 cells were seeded at a density of 1 × 10^4^ cells per well and 1.2 × 10^4^ cells per well in a 96‐well plate, respectively. After 24 h, the cells were administered increasing concentrations of nanoparticles, measured by iron‐oxide content (0, 40, 80, 120, 160, and 200 µg mL^−1^). At 24 h, AlamarBlue reagent (10%) was added to the cells. Absorbance was measured 6 h later at 570/600 nm using the EnSight plate reader. Nanoparticle uptake was measured in HCM3 and D425 cells seeded at a density of 1.5 × 10^5^ in a 6‐well plate overnight before being transfected with FNP1, FNP2, and NFNP nanoparticles in OptiMem. All cells were administered transfection reagent (1 mL) and maintained at 5% CO_2_ at 37 °C for 4 h before replacing with fresh media. The following day, the cells were lysed and sent for ICP analysis.

#### Methods: siRNA Transfection

Cells (D425, HCM3, MCF7, and HCT116) were plated at a seeding density of 1.5 × 10^5^ in a 6‐well plate overnight before being transfected with siRNA using a previously described protocol.^[^ ^51^ ^]^ Briefly, siRNA (100 nm) was complexed to the three nanoparticles (50, 150, or 200 µg mL^−1^) in OptiMem and incubated for 20 min at room temperature (25 °C) as a transfection reagent. All cells were administered transfection reagent (1 mL) and maintained at 5% CO_2_ at 37 °C for 4 h. After 4 h, the transfection reagent was replaced with DMEM/10% FBS. Analysis was performed 24–72 h post exposure. siRNA used in these investigations are illustrated in the table below.

**Table:** 

Name	siRNA sequence/ordering number	Supplier
Non‐targeting control (Ctrl) siRNA	D‐001810‐10‐50	Dharmacon (US)
Human PLK1 siRNA	Sense strand: 5′‐GAAGGAGUGUGAAAACUGCUU‐3′ Antisense strand: 5′PAGACUCAGGCGGUAUGUGCUU‐3′	Dharmacon (US)
AF647 siRNA	1 027 295	Qiagen (Germany)

#### Methods: Flow Cytometry

Following siRNA transfection where AF647 siRNA was complexed to the nanoparticles, flow cytometry was used to examine cellular uptake by fluorescence in medulloblastoma cells. In brief, D425 cells were transfected for 4 h. At 4 and 24 h after transfection, the cells were trypsinized and collected in PBS. Cellular uptake was measured using the BD FACSAria II.

#### Methods: Immunofluorescence Confocal Microscopy

Cellular uptake was further examined using confocal microscopy to identify AF647 labeled siRNA. Briefly, 4 and 24 h post‐transfection, the cells were fixed with 4% paraformaldehyde (PFA) and permeabilized with 0.01% Triton X‐100. Followed by staining with Hoechst (1 µg mL^−1^) and ActinRed 555 (2 drops per mL). Cells were subsequently imaged on the Leica SP8 microscope using a 40× objective lens (Australian National Fabrication Facility). Lysosomal escape was examined using LysoTracker Yellow HCK‐123 (50 nM) for 30 min at 24 h post transfection.

#### Methods: Exocytosis

To examine exocytosis hCMEC/D3 cells were seeded into a 6‐well tissue culture plate. The following day, cells were transfected as previously described with FNP1‐AF647 siRNA. Following 3 h of incubation, the transfection reagent was removed, and cells were washed with PBS before adding fresh media (1 mL). One, four, and twenty‐four hours later, the medium was collected. Simultaneously, at each time point, cells were collected by adding RIPPA buffer (1 mL). Medium and cell lysates were transferred into a black 96‐well plate in triplicate. The fluorescence AF647 intensity was read using the EnSight plate reader.

#### Methods: Time‐Lapse Imaging using Operetta

To examine cellular uptake over time, the Operetta CLS High Content Analysis System was used as per the manufacturer's guidelines. Briefly, HCM3 and hCMEC/D3 cells were plated at a seeding density of 3.75 × 10^4^ in a 24‐well plate and transfected with nanoparticle‐AF647 siRNA complexes the following day as previously described. After 4/24 h transfection, images were collected every 30 min over a 15 h period. Cells were maintained at 5% CO_2_ at 37 °C during the image acquisition. ROS experiments were performed in these cells using CellROX Orange as per manufacturer guidelines (Thermo Fisher Scientific Inc).

#### Methods: Western Blotting

The block in PLK1 protein expression was examined using western blotting techniques. Briefly, Cells were transfected and collected at 24, 48, and 72 h post‐transfection for protein extraction. Protein quantification was by Peirce BCA assay following manufacturer guidelines. Protein samples (10 µg) in loading buffer were heated at 95 °C for 5 min and electrophoresed (120 V) for 1 h, followed by effective transfer to the membrane at 200 mA for 1 h. PLK1 protein expression was measured using a goat‐anti‐rabbit PLK1 antibody (1:1000; 5% BSA/TBST) (Cell signaling). Goat‐anti‐mouse β‐Actin (1:10000; 5% milk/TBST) was used as a control (Thermofisher). Primary antibodies were incubated with the membrane overnight at 4 ⁰C and for 1 h at room temperature (RT), respectively. Secondary antibodies for rabbit and mouse (1:10000; 5% milk/TBST) were incubated at RT for 1 h (Agilent). Clarity Max ECL was used to visualize the bands and imaging was performed using the GelDoc Go (Biorad).

#### Methods: Transwell BBB Model

To model the BBB, 1 × 10^4^ hCMEC/D3 or HUVEC cells were seeded onto the typical side of a transwell insert with a 0.4 µm pore size and 0.33 cm^2^ surface area. The cells were grown for ≈7 days prior to experiments with media changes every two days. No nanoparticle experiments were performed until transendothelial electrical resistance (TEER) values were stable and permeability assays confirmed proficient barrier integrity.

#### Methods: Characterization of BBB Model

The TEER was examined as a measure of BBB integrity. TEER analysis was performed using the EVOM3 instrument as per manufacturer guidelines. Briefly, each day three measurements were taken per insert and the results averaged. To calculate the unit area resistance (Ω cm^2^), the average number recorded was multiplied by the surface area of the insert (0.33 cm^2^).

The apparent permeability (Papp) as a measure of barrier integrity was measured using Albumin‐FITC using a previously described method.^[^ ^52^ ^]^ Briefly, the insert containing the BBB endothelial cells was exposed to Albumin–fluorescein isothiocyanate conjugate (A‐FITC MW 6.6 kDa) (10 µg mL^−1^) for 1 h at 37 °C. Fluorescence was measured using the EnSight plate reader. The permeability for both molecules was calculated from the formula:

(1) $$Papp\;=\;V_{r}C_{1}/t\;\times \;1/\left(SC_{0}\right)$$

where *Papp* is the apparent permeability, *V_r_* (mL) is the volume of medium in the receiver chamber, *C*_0_ (µg mL^−1^) is the initial concentration of fluorescent compound in the donor chamber, *S* (cm^2^) is the surface area of the monolayer, *C*_1_ (µg mL^−1^) is the concentration of fluorescent molecule in the receiver chamber after 1 h of incubation and *t* (s) is the incubation time. The apparent permeability coefficient was expressed in cm s^−1^.

Western blotting was performed as described above, using a ZO‐1 primary antibody (1:100 5% BSA/TBST) overnight at 4 °C (Thermo Fisher Scientific Inc) to determine the presence of tight junctions.

#### Methods: Nanoparticle‐siRNA Transcytosis

Once all characterizations had shown that the BBBs were fully functional, the nanoparticle‐AF647 siRNA complexes (200 µg mL^−1^; 100 nM) were added to BBB containing inserts positioned within a 24‐well receiver plate seeded (3.75 × 10^4^) with D425 in DMEM (no phenol red) and incubated for 24 h at 37 ⁰C. The following day the media in the receiver chamber was collected and fluorescence was measured using the EnSight plate reader. The D425 cells were fixed and stained as described above and imaged using the Leica SP8 microscope.

#### Methods: BBB Transcytosis and Uptake by Cancer Cells

To determine transcytosis followed by uptake by D425 cells, D425 cells were seeded (3.75 × 10^4^) in a 24‐well imaging receiver chamber. Nanoparticle‐AF647 siRNA complexes (200 µg mL^−1^; 100 nM) were added to BBB‐containing inserts positioned within a 24‐well receiver plate containing DMEM (no phenol red) and incubated for 24 h at 37 ⁰C. The following day the D425 cells were fixed and stained as described above and imaged using the Leica SP8 microscope.

#### Methods: Calcein Assay to Examine Endosomal Rupture

To examine whether the FNP1‐AF647 siRNA complexes were able to promote endosome rupture, calcein dye (150 µg mL^−1^) was mixed into transfection reagent pH 6.7 before adding to D425 and HCM3 cells. Live cell confocal microscopy was used to determine whether endosomal rupture had occurred 1–3 h. Calcein is a membrane‐impermeable fluorescent dye taken up by endosomes when nanoparticles are endocytosed. Inside the endosomes, the high local concentration of dye and low pH partially quenches the fluorescence of the dye. At this time calcein has a punctate distribution within the cell. If the membrane of the endosome ruptures, calcein travels into the cytosol and becomes an intense diffuse fluorescence.

#### Methods: Chelator‐Free Radiolabeling of Nanoparticles

The chelator‐free method of radiolabeling polymer nanoparticles was used.^[^ ^53^ ^]^ 20% Na_2_CO_3_ (1 m, pH ≈11) was added to Zirconium (^89^Zr) in oxalic acid solution (0.05 m) (Austin Health, Melbourne, Australia) to adjust the pH between 9 to 10. Equal volume of HEPES buffer (0.5 m, pH 7) was added to the solution to adjust the final pH to 7 for optimal labeling efficiency. The required volume was pipetted into a reaction vial. NFNP, FNP1, and FNP2 (5 µg per MBq) were added to the ^89^Zr solution for radiolabeling, at pH ≈7. The reaction mixture was incubated for 60 min at 75 °C in a thermomixer (Eppendorf) rotating at 400 rpm, as per previous experimental protocols undertaken.^[^ ^54^ ^]^

#### Methods: Radio (Instant) Thin Layer Chromatography

Radiolabeling efficiency was analyzed by Radio‐iTLC to detect unbound ^89^Zr. After heating, the reaction mixture (2 µL) was added to Diethylenetriaminepentaacetic acid (DTPA) (50 mm, 5 µL) and incubated for 3–4 min at room temperature. A control reaction was undertaken with ^89^Zr and DTPA independently. The resultant reaction mixture (1 µL) was spotted on iTLC‐SG glass macrofibre chromatography paper, embedded with silica gel (Agilent), and placed in H_2_O/EtOH (50:50, v/v). Solvent traveled up the strip via capillary action and was removed when reached ¾ of the strip. Spotted iTLC strips were airdried, allowing for solvent to evaporate before being analyzed to detect particle bound ^89^Zr‐NFNP, ^89^Zr‐FNP1, ^89^Zr‐FNP2 (R_f_ = 0) and DTPA chelated ^89^Zr (R_f_ = 1) in the reaction mixture. Analysis was performed on a Radio‐iTLC mini scanner and flow count imaging scanner (B‐MS‐1000F, Eckert & Ziegler) measuring the radiolabeled nanoparticle retention factor, expressed as a percentage.

#### Methods: Dose Preparation

Each ^89^Zr nanoparticle formulation (20 MBq) was diluted in 1× PBS (1 mL) and individually drawn up into syringes and calibrated (Capintec CRC‐25 Dose Calibrator) prior to injection. The injected volume was not >200 µL per mouse. Mice were injected while in the PET‐CT scanner and the residual activity remaining in the syringe and cannula was measured to determine injected dose.

#### Methods: PET‐CT Imaging

All studies were in accordance with guidelines of the Animal Ethics Committee of The University of Queensland (UQ; Approval 2020/AE000044) and the Australian Code for the Care and Use of Animals for Scientific Purposes. Female C57/BL6J mice (≈8 weeks of age) were acquired from the Animal Resource Centre (Western Australia) and housed in temperature and humidity‐controlled housing with ad libitum access to food and water.

The imaging study was divided into three sample groups of non‐tumor‐bearing, mice; i) ^89^Zr‐NFNP (n = 2), ii) ^89^Zr‐FNP1 (n = 3), and iii) ^89^Zr‐FNP2 (n = 2). The mice were anaesthetized (2% isoflurane, 1–2 L min^−1^ O_2_) via inhalation, for cannulation of the lateral tail vein and imaging. Mice were positioned prone, in a prefabricated holder to prevent movement and improve imaging co‐registration. Imaging was performed on a Siemens Inveon PET/CT scanner. Dynamic acquisition commenced 1 min prior to intravenous injection of ^89^Zr nanoparticle (3‐5 MBq), to assess dynamic cardiac and systemic perfusion over 45 min. Subsequent 4 and 24 h post‐injection whole body, static imaging was performed. Upon completion of each PET image, a 12 min micro CT was acquired for attenuation correction and anatomical co‐registration. CT was performed with an X‐ray tube potential and current of 80 kV and 500 µA, respectively. 360° rotation in 120 rotational steps permitted 3D imaging, with a low magnification and binning factor of 4 was used. A respiratory probe was used throughout imaging study to perform physiological monitoring (BioVet system, m2m Imaging, Australia). PET data reconstruction was processed using the Ordered Subset Expected Maximum (OSEM2D) algorithm on Inveon Research Workspace software (IRW v4.1, Siemens, Germany).

Co‐registration was achieved by manual and automatic alignment of CT images with corresponding PET signals. Hybrid fusion Maximum Intensity Projection (MIP) images were produced for 3D visualization of the mouse's physiological uptake of the radiolabeled nanoparticle. Inveon Research Workplace was used to draw volumetric regions of interest within the whole body and organs of interest; heart, liver, spleen, kidneys, bladder, spleen, and using morphologically guided CT to delineate organs. Activity per voxel was converted to MBq mL^−1^ using a conversion factor, obtained by scanning a cylindrical filled phantom with a known activity of ^89^Zr, to calibrate scanner efficiency. Activity concentrations were decay corrected and expressed as a percentage of injected activity per cm^3^ of tissue, approximating a percentage injected dose/gram (%ID g^−1^).

#### Methods: Ex vivo Tissue Analysis for Biodistribution

Following 24 h imaging, and still under anesthesia, blood collection was achieved by cardiac puncture followed by euthanasia. Organs/tissues (liver, spleen, heart, kidneys, brain, bone, gastrointestinal tract, and tail) were extracted, and cleaned of excess blood. All samples were weighed for ex vivo radioactivity analysis. Using a gamma scintillation counter (Wizard 2480 Automatic Gamma Counter, PerkinElmer), ^89^Zr activity retained in the sampled tissues was counted, and calibrated to a known ^89^Zr activity prior to measurement. Measured ^89^Zr activity from all samples was calculated with the sample weight and injected dose, to determine the percentage of injected dose per gram of the sampled tissue/organ (%ID g^−1^), expressed as a mean ± standard deviation.

#### Methods: MR Imaging

All studies were in accordance with guidelines of the Animal Ethics Committee of The University of Queensland (UQ; Approval 2021/AE000666) and the Australian Code for the Care and Use of Animals for Scientific Purposes. Female Balb/c nude mice (6 weeks of age) were acquired from the Animal Resource Centre (Western Australia) and housed in temperature and humidity‐controlled housing with ad libitum access to food and water.

An orthotopic medulloblastoma model was developed using human medulloblastoma cell line D425 transduced to express GFP/luciferase (GenTarget, US). For tumor implantation, 1 × 10^5^ D425 cells in 80% Matrigel (2 µL) were injected in the right cerebellum of 6‐week‐old female Balb/c nude mice using a Hamilton syringe. One week after tumor plantation, the time‐dependent MR images were assessed in 3 mice, captured on 7.0 T animal MRI scanner (Burker ClinScan, Germany). In detail, the mice were anesthetized (1–2% isoflurane, 2 L min^−1^ O_2_), and then tail intravenously injected with 10 mg kg^−1^ nanoparticle. *T*_1_‐ and *T*_2_‐weighted MR images were obtained using a modified multiple spine echo sequence before and at different time points after injection. The signal intensity and *T*_1_, and *T*_2_ of the tumor from the cerebellum were measured by Image J and Paravision 360 v3.4, respectively. The imaging parameters were set as follows: *T*_2_‐weighted imaging: TR = 2600 ms, TE = 34.5 ms, matrix = 192 × 192, FA = 180, slice thickness = 0.600 mm. *T*_1_‐weighted imaging: TR = 700 ms, TE = 8.4 ms, matrix = 320 × 256, FA = 180, slice thickness = 0.600 mm. To calculate *T*_2_ times, the following parameters were used: TR = 5500 ms, with multiple TE = 8.5, 25.5, 42.5, 59.5, 76.5, and 93.5 ms, matrix = 160 × 160, FA = 180, slice thickness = 0.800 mm. To calculate *T*_1_ times, multiple TR = 5500, 3000, 1500, 800, and 450 ms were acquired for each echo time.

#### Methods: In Vivo FNP1‐siRNA Effects on Growth

Two treatment regimens were investigated, a 3‐day consecutive and an 8‐day schedule where mice were administered FNP1‐PLK1 siRNA every other day (×4 total treatments). As per ethics approval 2021/AE000666, mice with established medulloblastoma as described in 4.2.29 were placed into treatment groups with an even tumor size distribution using IVIS (PerkinElmer, US) measurements of luciferase. The control group (n = 3) received FNP1 only (10 mg kg^−1^), whilst the treatment group (n = 4 for 3‐day; n = 5 for 8‐day) received FNP1‐PLK1 siRNA (10 mg kg^−1^:3 mg kg^−1^, respectively). Examination of growth during and after treatment was done using IVIS to measure luciferase expression.

#### Methods: Histopathological Examination

Brains were harvested and fixed with 4% PFA for 24 hours at 4 ⁰C, embedded in paraffin wax, and sectioned (thickness = 10 µm). The slices were deparaffinized and rehydrated. For H&E staining, the slices were stained with Mayers hematoxylin for 3 min, washed with water, and differentiated with 1% sodium bicarbonate solution. After that, the slices were incubated with eosin for 10 s, washed with water, and then dehydrated with alcohol. Finally, the slices were sealed with xylene and mounted. H&E slices were imaged with an Axio Imager Azure microscope (Zeiss, Germany).

#### Statistical Analysis

Data were presented as the mean ± the standard error of the mean (SEM) unless otherwise stated. Data was characterized using the Student's *t*‐test, one sample *t*‐test, one‐way ANOVA, Tukey's multiple comparisons test, two‐way ANOVA, Dunnett's multiple comparisons test, and Wilcoxon test using GraphPad Prism 9.4 software. The *p*‐value of ≤0.05 (*) was considered significant.

## Conflict of Interest

The authors declare no conflict of interest.

## Author Contributions

H.F., J.Z., and X.H. contributed equally to this work. T.P.D. and R.Q. performed conceptualization; T.P.D., R.Q., K.T., H.F., X.H., J.Z., C.Z., N.F., and G.C. performed methodology; H.F., X.H., J.Z., C.Z., L.L., Y.C.W., N.F., H.B., G.C., K.M., and J.H. performed investigation; X.H., J.Z., H.F., H.B., and J.H. performed visualization; T.P.D., R.Q., K.T., and N.F. did supervision; R.Q., H.F., X.H., J.Z., and H.B. wrote original draft; T.P.D., R.Q., K.T., H.F., X.H., J.Z., C.Z., N.F., G.C., L.L., K.M., J.H., and M.K. wrote the original draft and reviewed and edited the final manuscript.

## Supporting information

Supporting Information

Supplemental Movie 1

Supplemental Movie 2

Supplemental Movie 3

## Data Availability

The data that support the findings of this study are available from the corresponding author upon reasonable request.
