# Supplementary material for: Multifunctional Fluoropolymer‐Engineered Magnetic Nanoparticles to Facilitate Blood‐Brain Barrier Penetration and Effective Gene Silencing in Medulloblastoma
Source: Adv Sci (Weinh). 2024 Apr 22;11(25):2401340. doi: 10.1002/advs.202401340 (PMC11220643; doi:10.1002/advs.202401340)
Supplement: Supplementary file 1 — Supporting Information [file ADVS-11-2401340-s002.docx]

Supplementary Materials for

**Multifunctional Fluoropolymer-Engineered Magnetic Nanoparticles to Facilitate Blood-Brain Barrier Penetration and Effective Gene Silencing in Medulloblastoma**

*Helen Forgham,† Jiayuan Zhu,† Xumin Huang,† Cheng Zhang, Heather Biggs, Liwei Liu, Yi Cheng Wang, Nicholas Fletcher, James Humphries, Gary Cowin, Karine Mardon, Maria Kavallaris, Kristofer Thurecht, Thomas P. Davis,^*^ Ruirui Qiao^*^*

*Corresponding author. Email: [t.davis@uq.edu.au](mailto:t.davis@uq.edu.au); r.qiao@uq.edu.au

**This PDF file includes:**

Figures. S1 to S19

Tables S1 to S4

**Other Supplementary Materials for this manuscript include the following:**

Movies S1-S3

Supplementary Text

^1^H NMR spectra


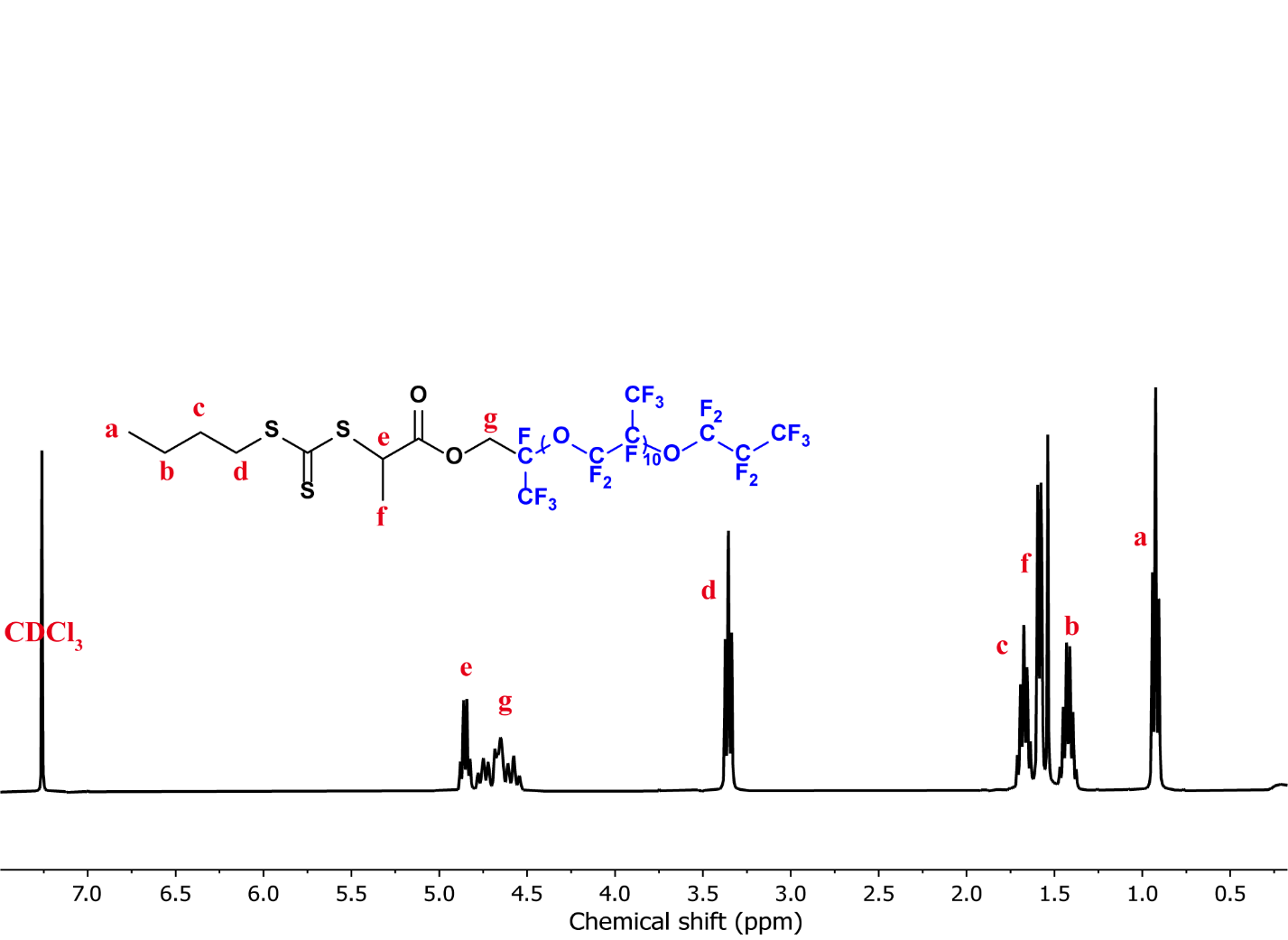
Figure S1.

The ^1^H NMR spectra of PFPE-CTA in CDCl_3_.

^19^F NMR spectra
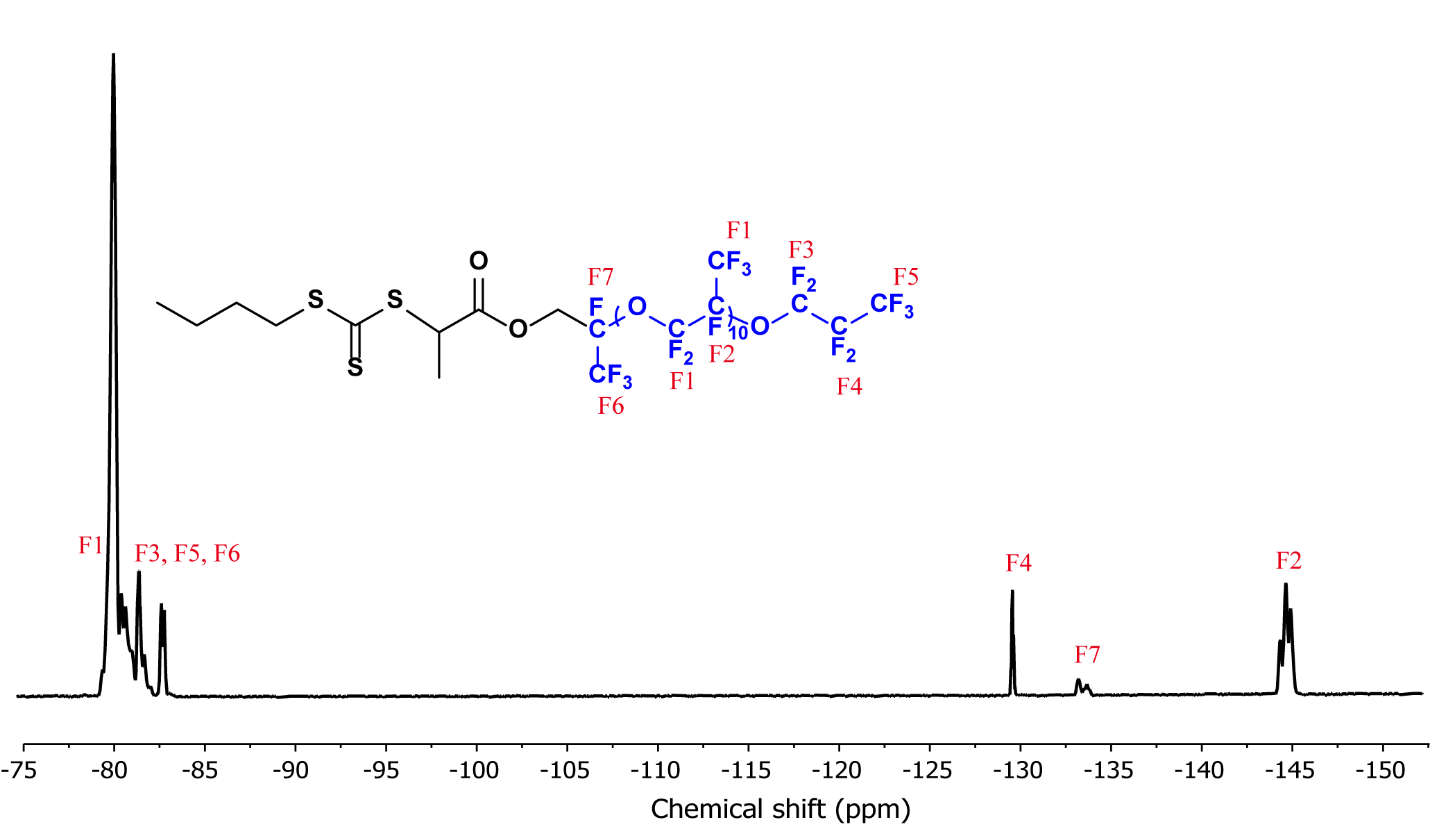


Figure S2.

The ^19^F NMR spectra of PFPE-CTA in CDCl_3_.

^1^H NMR spectra


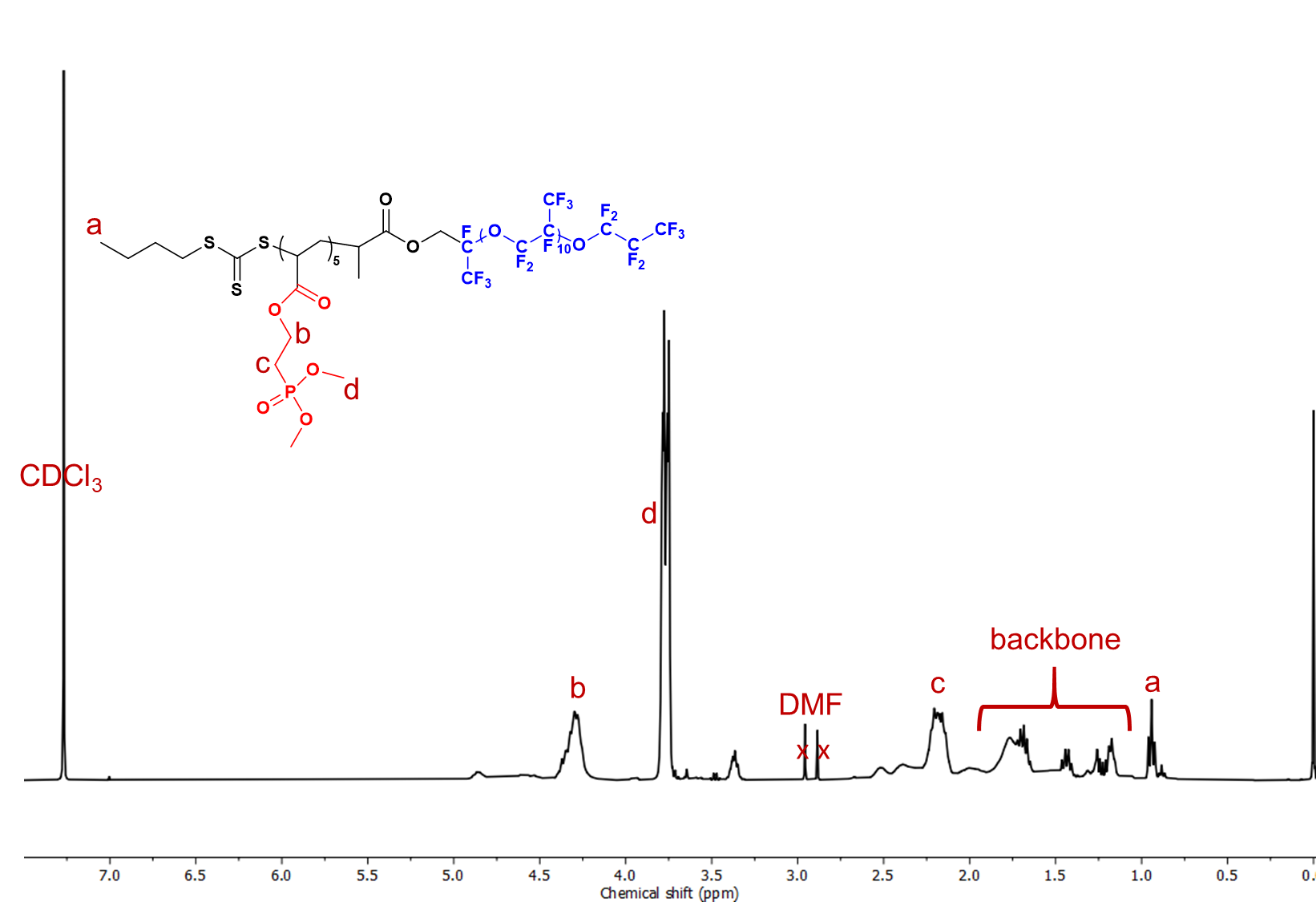


Figure S3.

The ^1^H NMR spectra of PFPE-PA_6_ in CDCl_3_.

^1^H NMR spectra


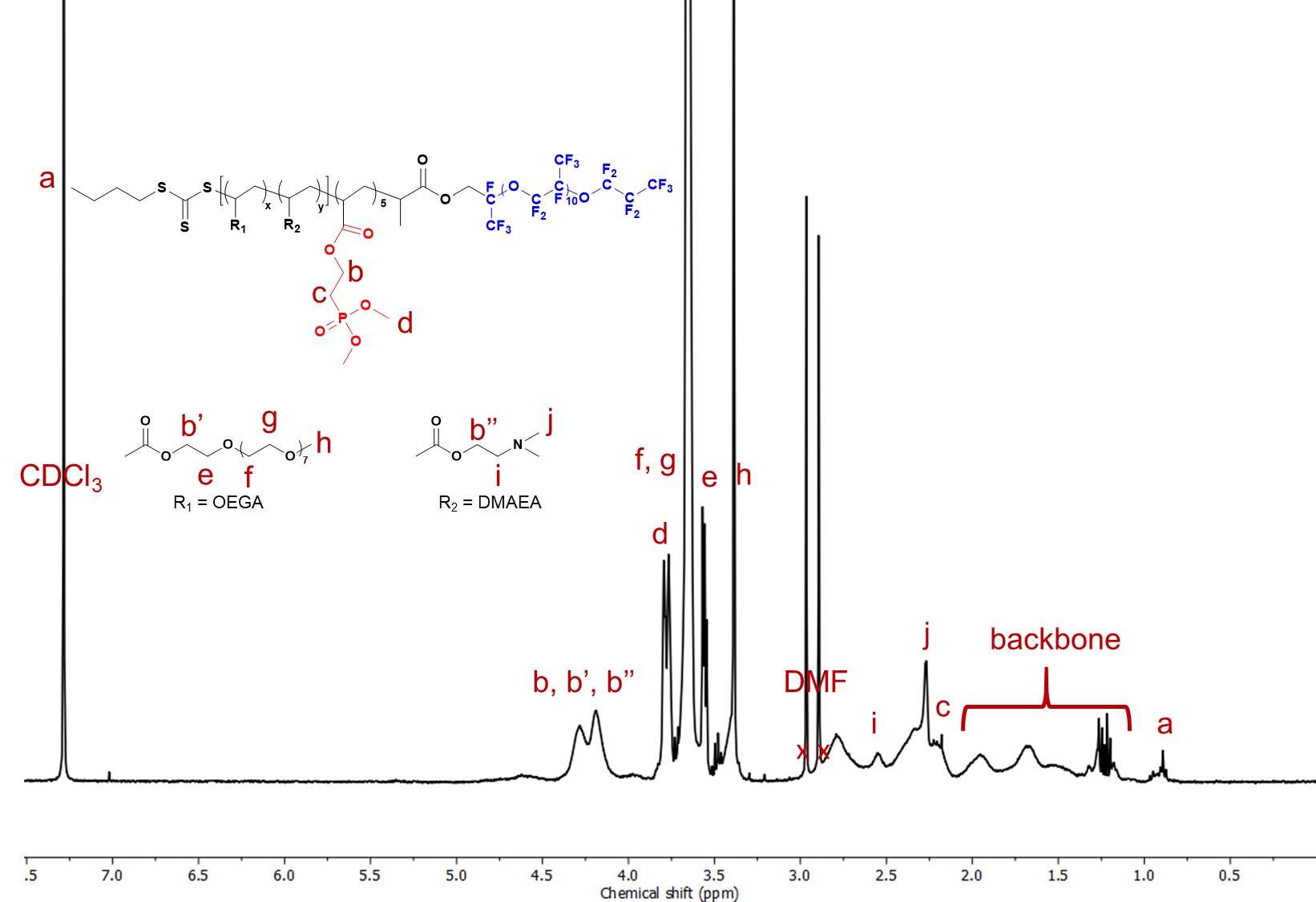


Figure S4.

The ^1^H NMR spectra of PFPE-PA_6_-*b*-(OEGA_12_-*co*-DMAEA_6_) in CDCl_3_.

^1^H NMR spectra


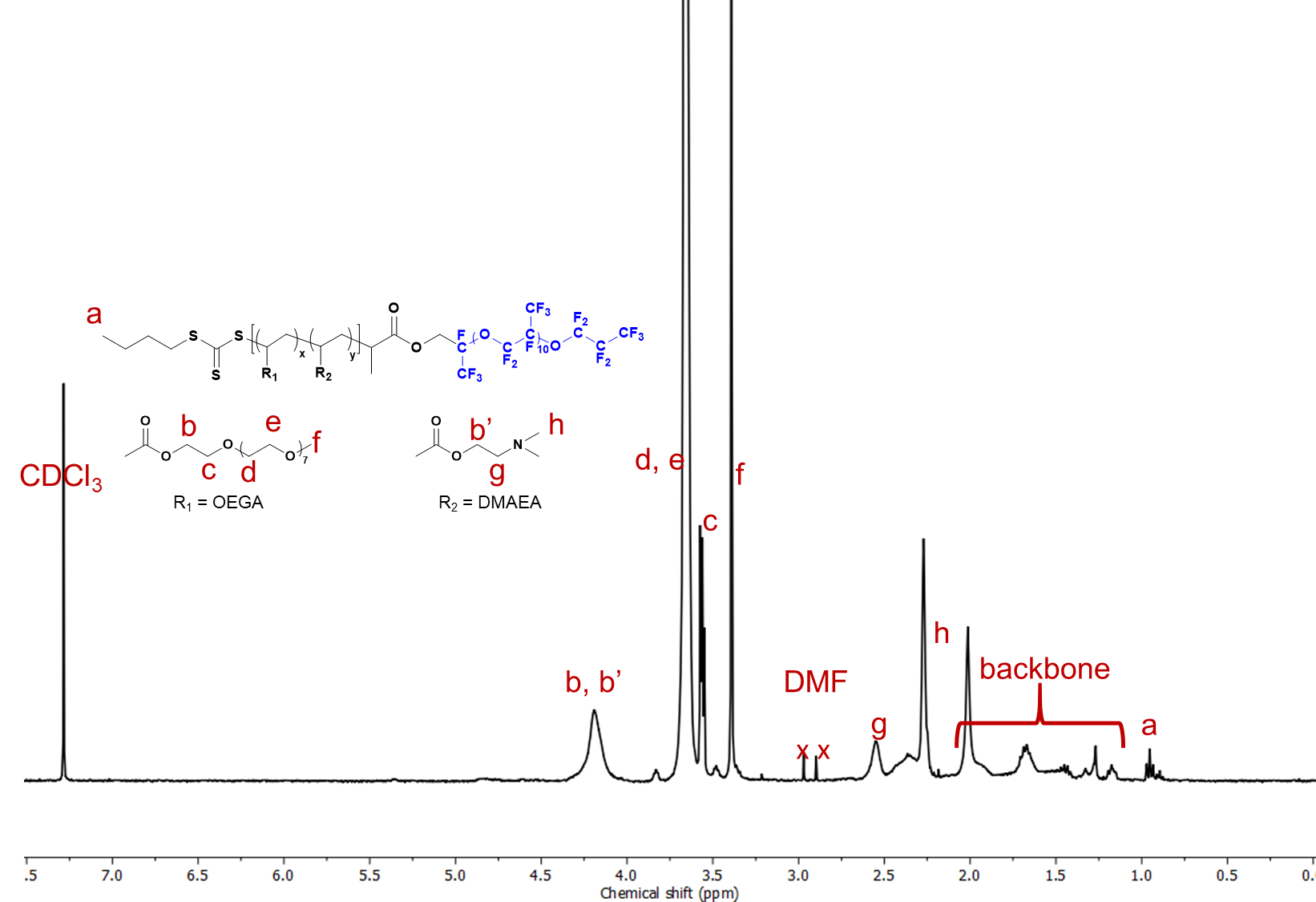


Figure S5.

The ^1^H NMR spectra of PFPE-(OEGA_11_-*co*-DMAEA_6_) in CDCl_3_.

^1^H NMR spectra


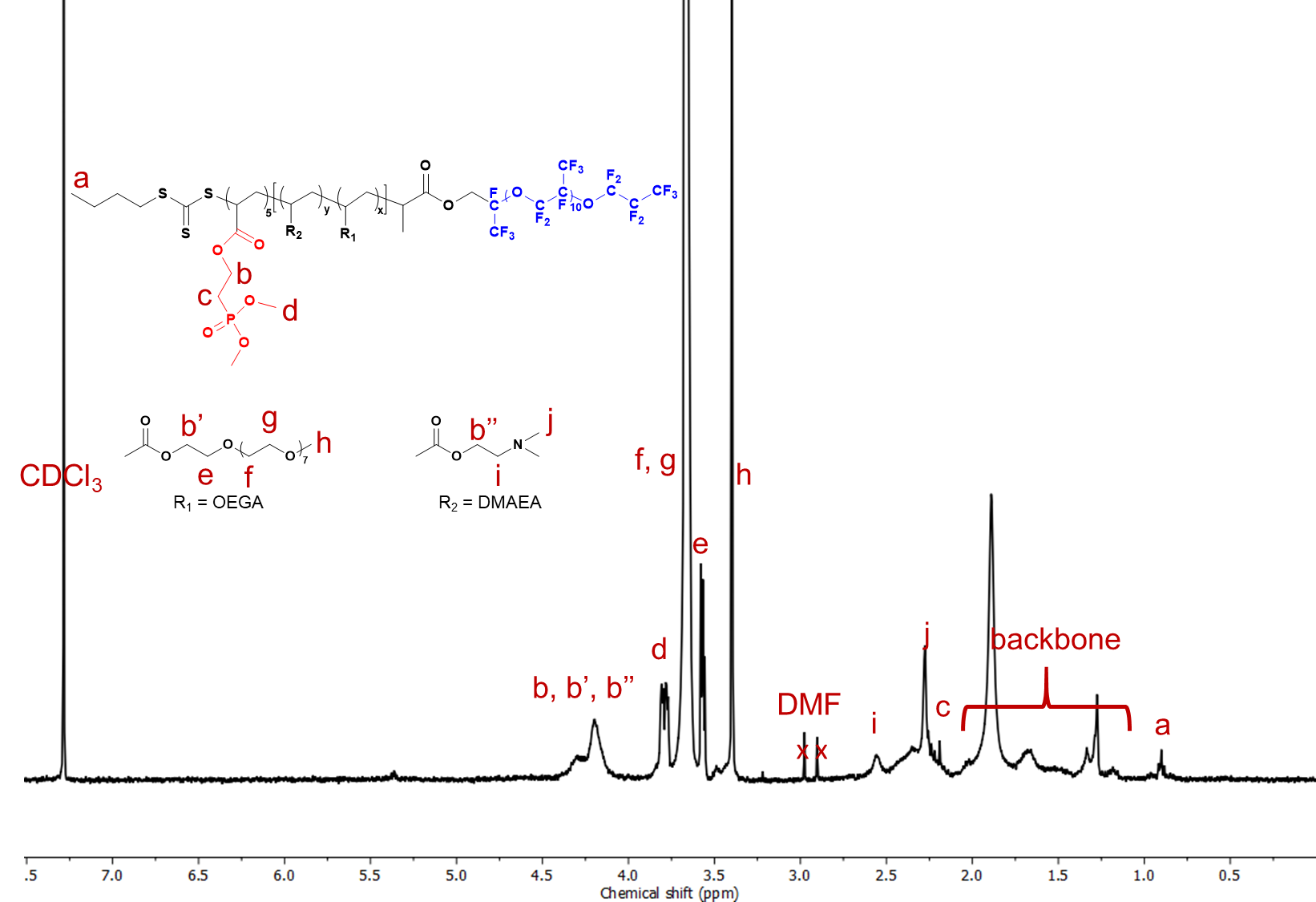


Figure S6.

The ^1^H NMR spectra of PFPE-(OEGA_11_-*co*-DMAEA_6_)-*b*-PA_5_ in CDCl_3_.

^1^H NMR spectra


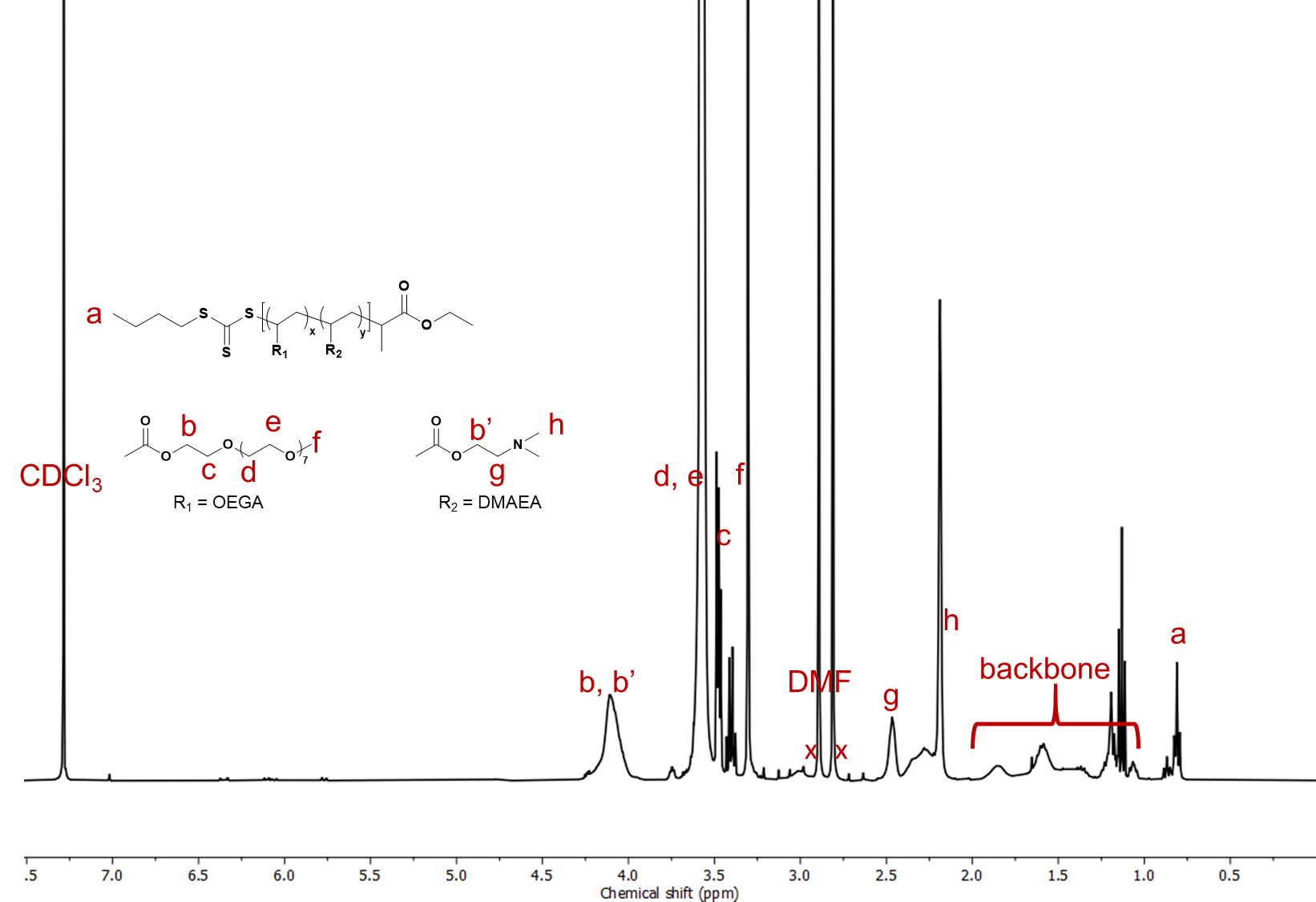


Figure S7.

The ^1^H NMR spectra of BTPA-(OEGA_11_-*co*-DMAEA_5_) in CDCl_3_.

^1^H NMR spectra


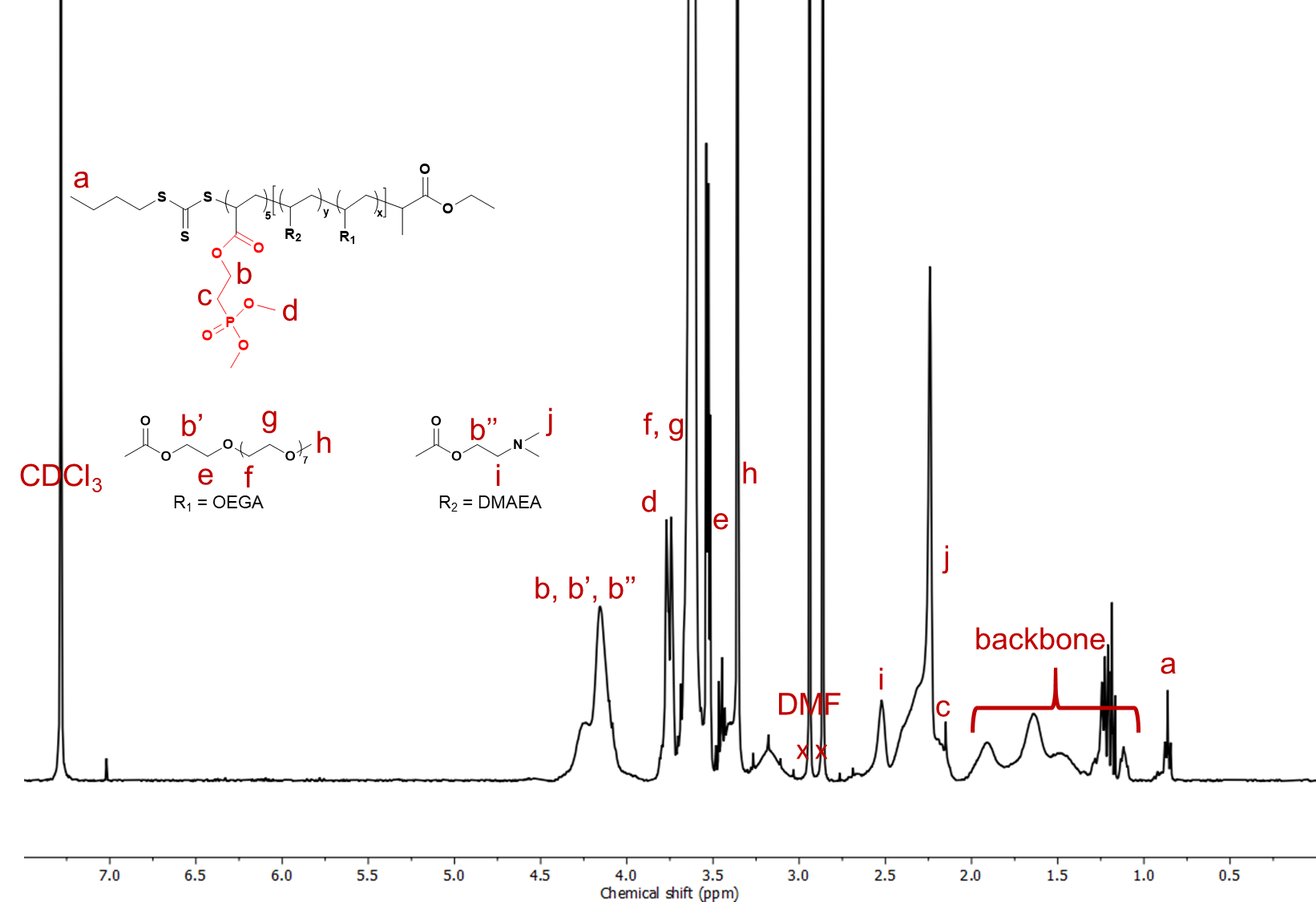


Figure S8.

The ^1^H NMR spectra of BTPA-(OEGA_11_-*co*-DMAEA_5_)-*b*-PA_8_ in CDCl_3_

Table S1.

Summary of various polymer preparation for siRNA delivery *via* RAFT polymerization.

| Entry | Name | Ratio of OEGA to CTA | Ratio of DMAEA to CTA | Ratio of PA to CTA | Reaction Time (h) | Conversion Rate | ^a^DP | ^a^M_n_,_NMR_  (g mol^-1^) | ^b^M_n,SEC_  (g mol^-1^) | ^b^M_w,SEC_  (g mol^-1^) | *Ð* |
| --- | --- | --- | --- | --- | --- | --- | --- | --- | --- | --- | --- |
| 1 | PFPE-PA_6_ |  |  | 5 | 5 | 92% | PA:6 | 3437 | N/A | N/A | N/A |
| 2 | PFPE-PA_6_-*b*-(OEGA_12_-*co*-DMAEA_6_) | 12 | 7 |  | 14 | 93% | OEGA/DMAEA:12/6 | 10056 | 6392 | 7542 | 1.18 |
| 3 | PFPE-(OEGA_11_-*co*-DMAEA_6_) | 12 | 7 |  | 3.5 | 88% | OEGA/DMAEA:11/6 | 8339 | 7050 | 7758 | 1.10 |
| 4 | PFPE-(OEGA_11_-*co*-DMAEA_6_)-*b*-PA_5_ |  |  | 6 | 14 | 98% | PA:5 | 9370 | 6822 | 7889 | 1.16 |
| 5 | BTPA-(OEGA_11_-*co*-DMAEA_5_) | 12 | 6 |  | 3.5 | 95% | OEGA/DMAEA:11/5 | 6222 | 6026 | 7224 | 1.20 |
| 6 | BTPA-(OEGA_11_-*co*-DMAEA_5_)-*b*-PA_8_ |  |  | 8 | 14 | 89% | PA: 8 | 7872 | 6630 | 7832 | 1.18 |

^a^Degree of polymerization (DP) was calculated from the integral of corresponded proton signals in ^1^H NMR spectra. ^b^Number average molecular weight was determined by DMF SEC using PS as standard.

Polydispersity of polymers


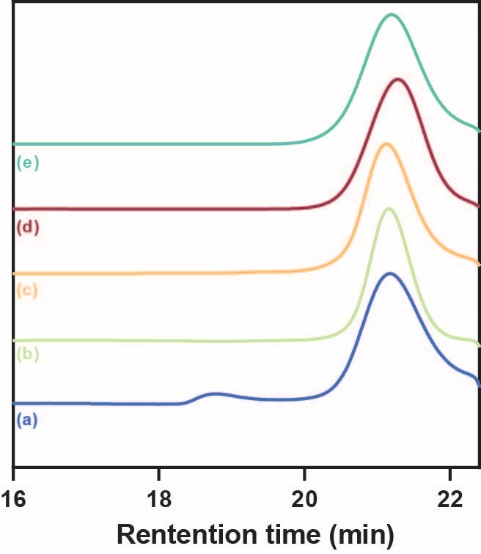


Figure S9.

Polydispersity of polymers characterized by size exclusion chromatography (SEC). SEC traces of (a) PFPE-PA_6_-*b*-(OEGA_12_-*co*-DMAEA_6_), (b) PFPE-(OEGA_11_-*co*-DMAEA_5_), (c) PFPE-(OEGA_11_-*co*-DMAEA_5_)-*b*-PA_5_, (d) BTPA-(OEGA_11_-*co*-DMAEA_5_), and (e) BTPA-(OEGA_11_-*co*-DMAEA_5_)-*b*-PA_8_.

TEM


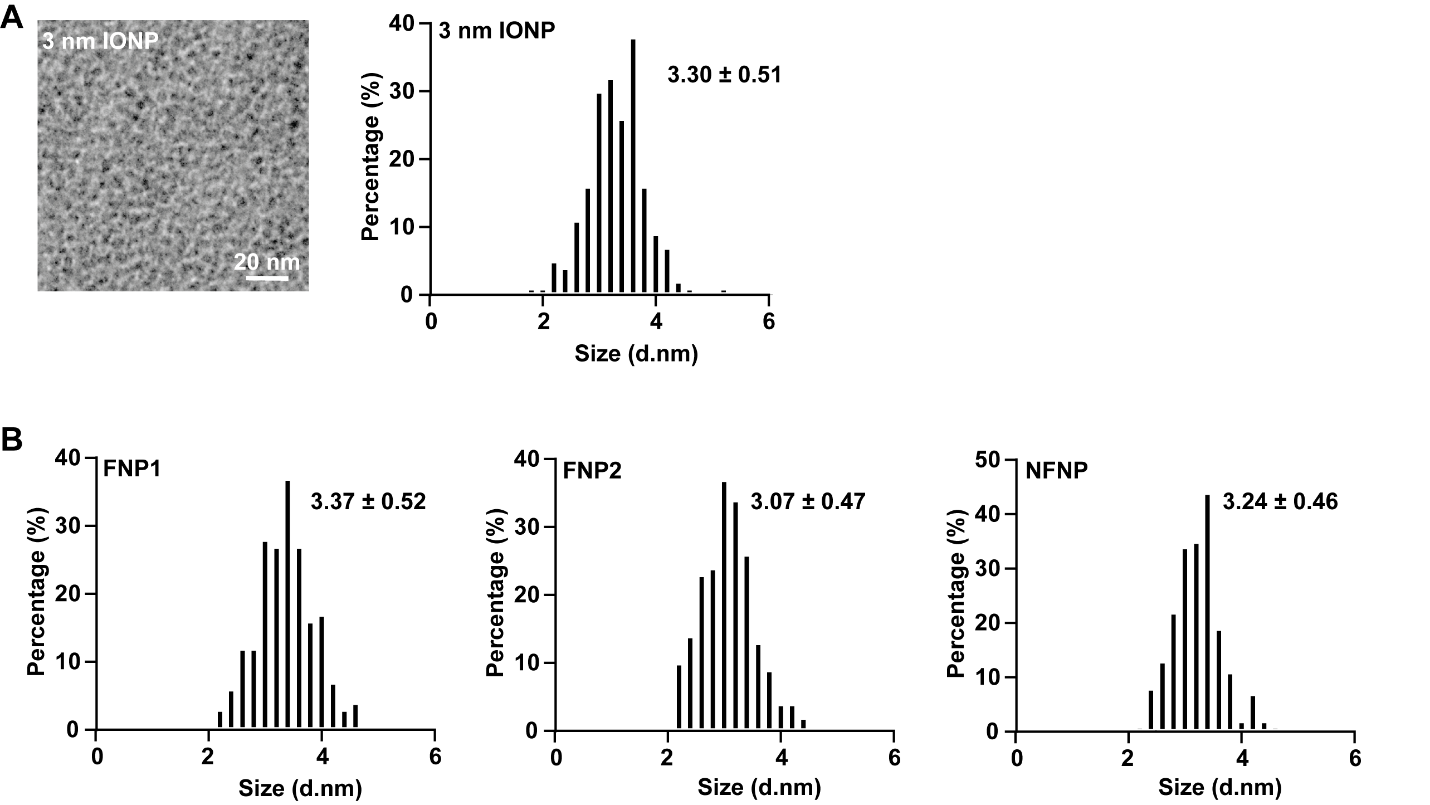


**Figure S10. TEM for 3 nm IONP**

(**A**) Representative TEM image and histogram of 3 nm IONP (scale bar: 20 nm). (B) Histogram details nanoparticle size variation for FNP1, FNP2 and NFNP.

FT-IR

**
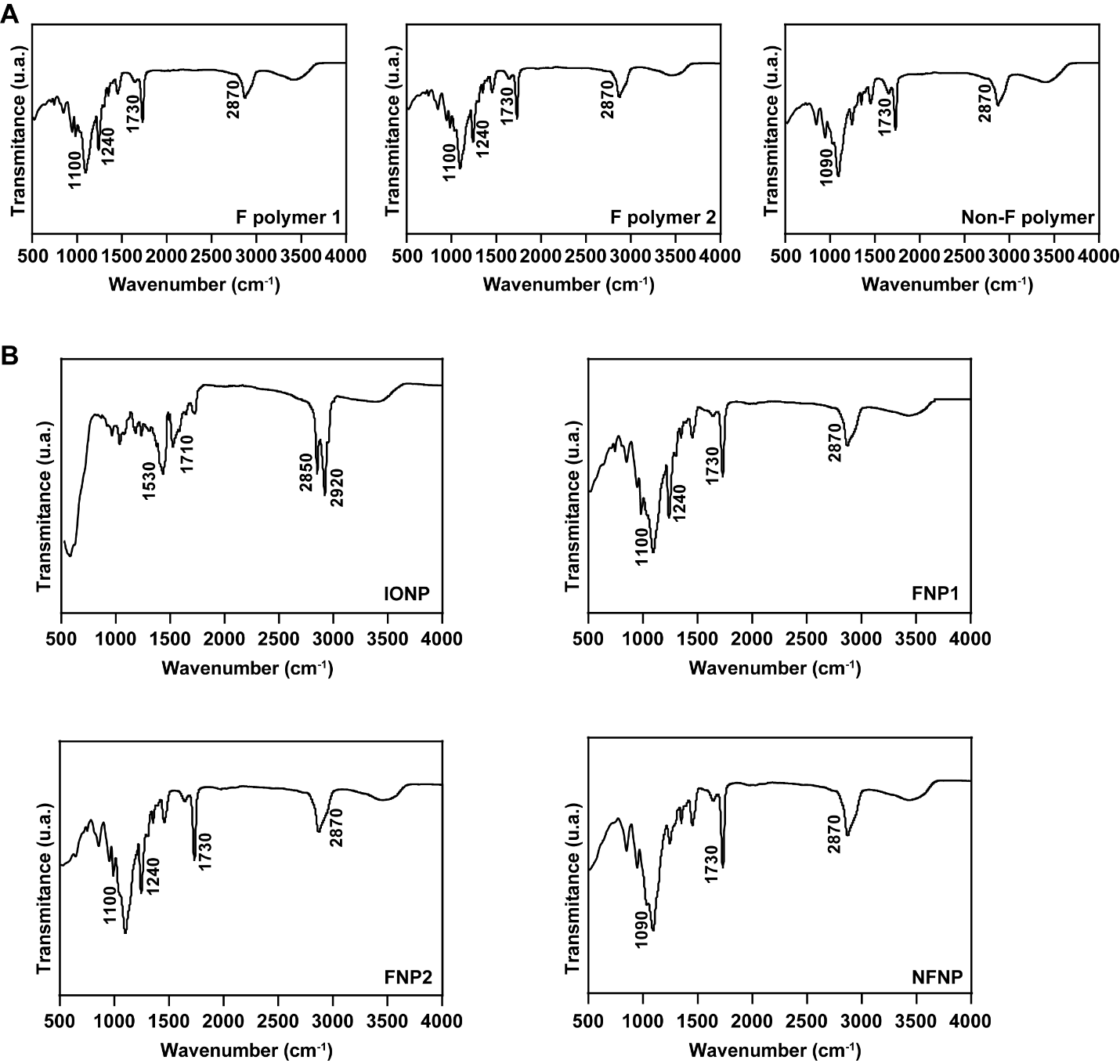
**

**Figure S11. FT-IR**

FT-IR spectra of (**A**) polymers: fluorinated polymer 1, fluorinated polymer 2 and non-fluorinated polymer (left to right). (**B**) nanoparticles: oleic acid coated IONPs, FNP1, FNP2 and NFNP. 1100 cm^-1^: C-O stretching, 1240 cm^-1^: C-N stretching, 1530: N-O stretching, 1710 and 1730 cm^-1^: C=O stretching, 2850-2920 cm^-1^: C-H stretching.

Table S2.

Relaxivity of nanoparticles in OptiMem by 7.0 T MRI.

| Nanoparticle | *r*_1_ (mM^-1^ s^-1^) | *r*_2_ (mM^-1^ s^-1^) | *r*_2_/*r*_1_ |
| --- | --- | --- | --- |
| FNP1 | 0.1352 | 8.054 | 59.57 |
| FNP2 | 0.6253 | 25.116 | 40.17 |
| NFNP | 0.1558 | 7.174 | 46.05 |

**
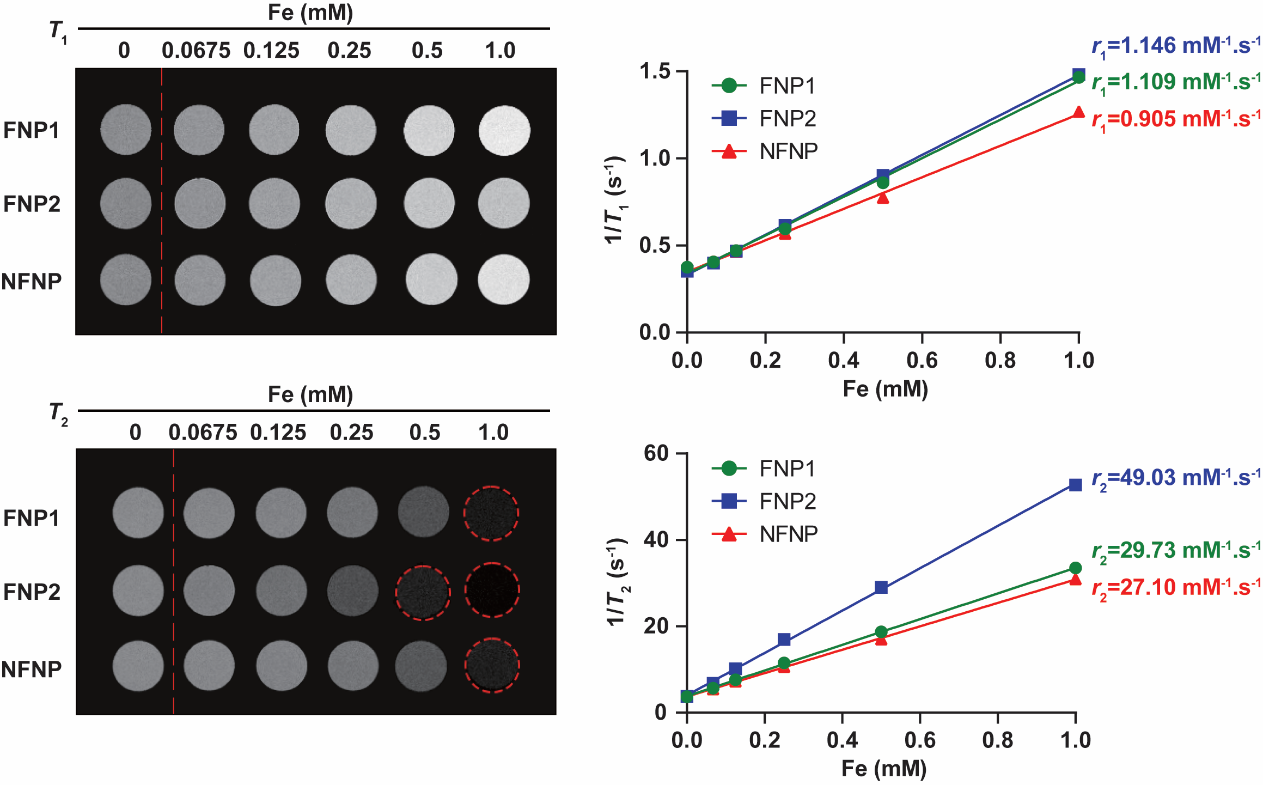
**

**Figure S12. MR imaging of iron oxide nanoparticles in H_2_O.**

Representative image demonstrating *T*_1_ and *T*_2_ of FNP1, FNP2 and NFNP in H_2_O against the concentration of Fe ion determined by 9.4 T MRI and quantified results as graphed data.

Table S3.

Relaxivity of nanoparticles in H_2_O by 9.4 T MRI.

| Nanoparticle | *r*_1_ (mM^-1^ s^-1^) | *r*_2_ (mM^-1^ s^-1^) | *r*_2_/*r*_1_ |
| --- | --- | --- | --- |
| FNP1 | 1.109 | 29.73 | 26.81 |
| FNP2 | 1.146 | 49.03 | 42.78 |
| NFNP | 0.905 | 27.10 | 29.94 |

**
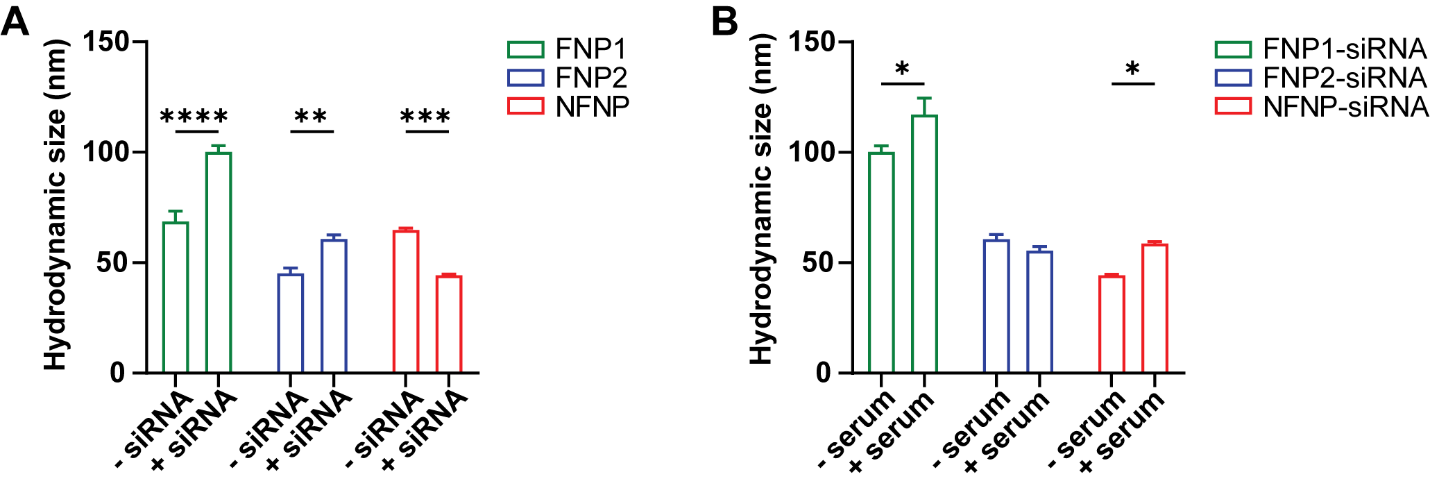
**

**Figure S13. Hydrodynamic size of FNP1, FNP2 and NFNP.**

Graph depicting the hydrodynamic size of FNP1, FNP2, and NFNP. (A) NP before and after complexed with siRNA, **p ≤ 0.01, ***p ≤ 0.001, ****p ≤ 0.0001, two-way ANOVA, Šídák's multiple comparisons test. (B) NP-siRNA complexes in DMEM with and without serum, *p ≤ 0.05, two-way ANOVA, Šídák's multiple comparisons test. All experiments described are n = 3.

**
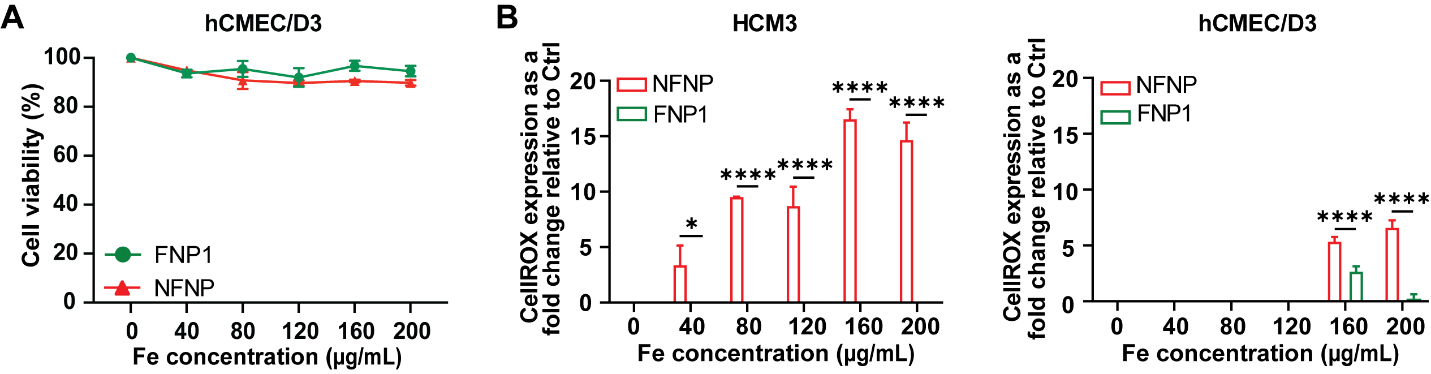
**

**Figure S14. ROS after incubation with FNP1 and NFNP.**

(A) Graphs illustrating the cytotoxic effect of nanoparticle vehicles on hCMEC/D3. (B) Graphs depicting CellROX expression as a marker for oxidative stress. NFNP were examined against fluorinated nanoparticles (FNP1), *p ≤ 0.05, ****p ≤ 0.0001, two-way ANOVA, Šídák's multiple comparisons test. All experiments described are n = 3.

Movie S1. Real-time imaging for internalization siRNA delivered by FNP1.
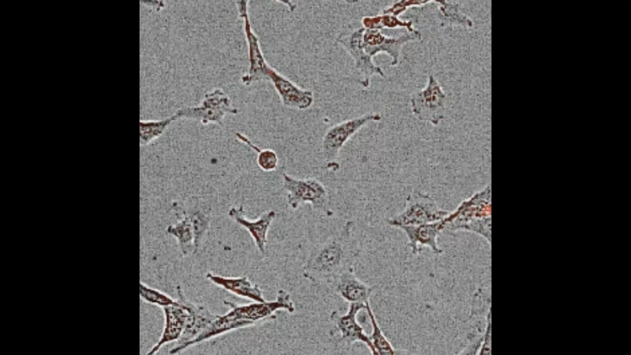


Movie S2. Real-time imaging for internalization siRNA delivered by FNP2.
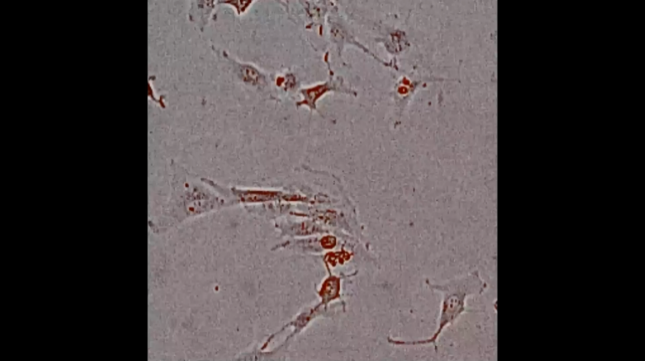


**Movie S3.** Real-time imaging for internalization siRNA delivered by NFNP.
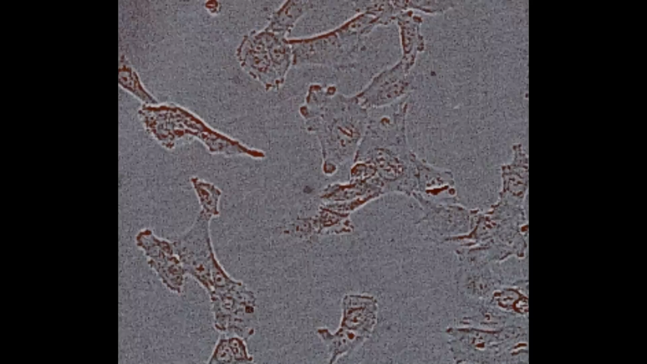


Cellular uptake


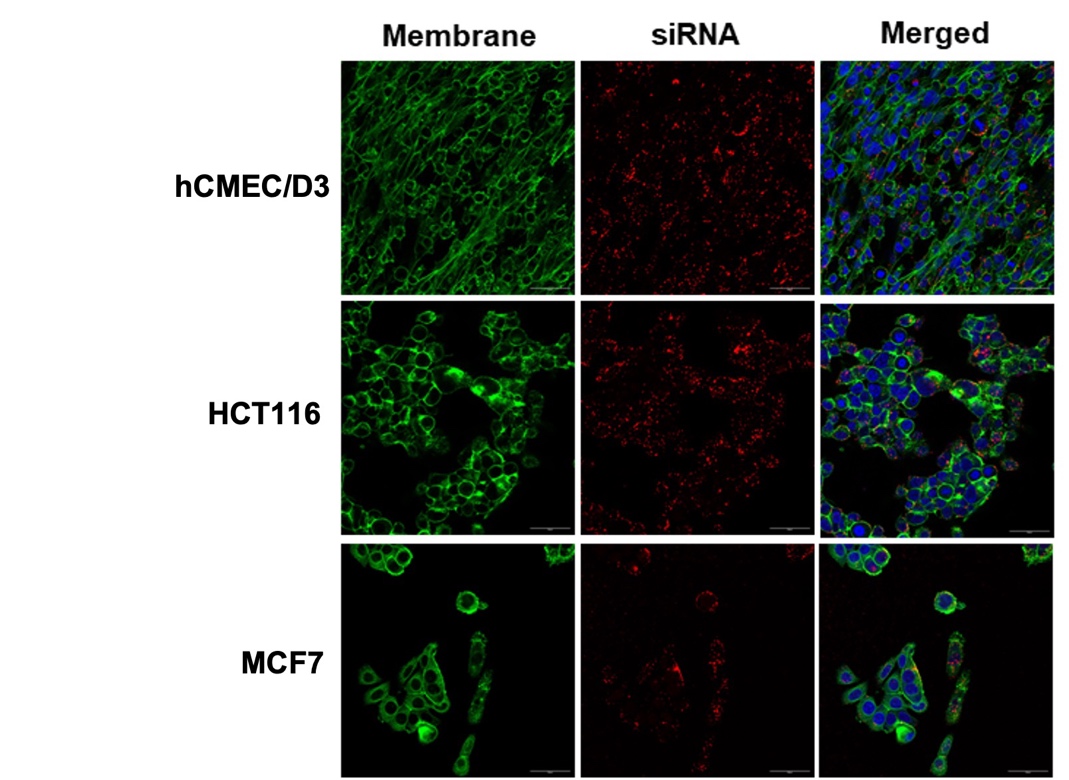


Figure S15. Cellular uptake

Representative confocal microscopy images showing the cellular uptake of siRNA (red) complexed to FNP1 (scale bar: 50 µm) in different cell lines.

Table S4. TLC radiochromatography.

| Nanoparticle | Identifier | Area | % Peaks |
| --- | --- | --- | --- |
| FNP1 | Peak 1 | 5927.50 | 100.00 |
| FNP2 | Peak 1 | 4736.50 | 97.00 |
|  | Peak 2 | 146.50 | 3.00 |
| NFNP | Peak 1 | 6732.50 | 98.85 |
|  | Peak 2 | 78.50 | 1.15 |


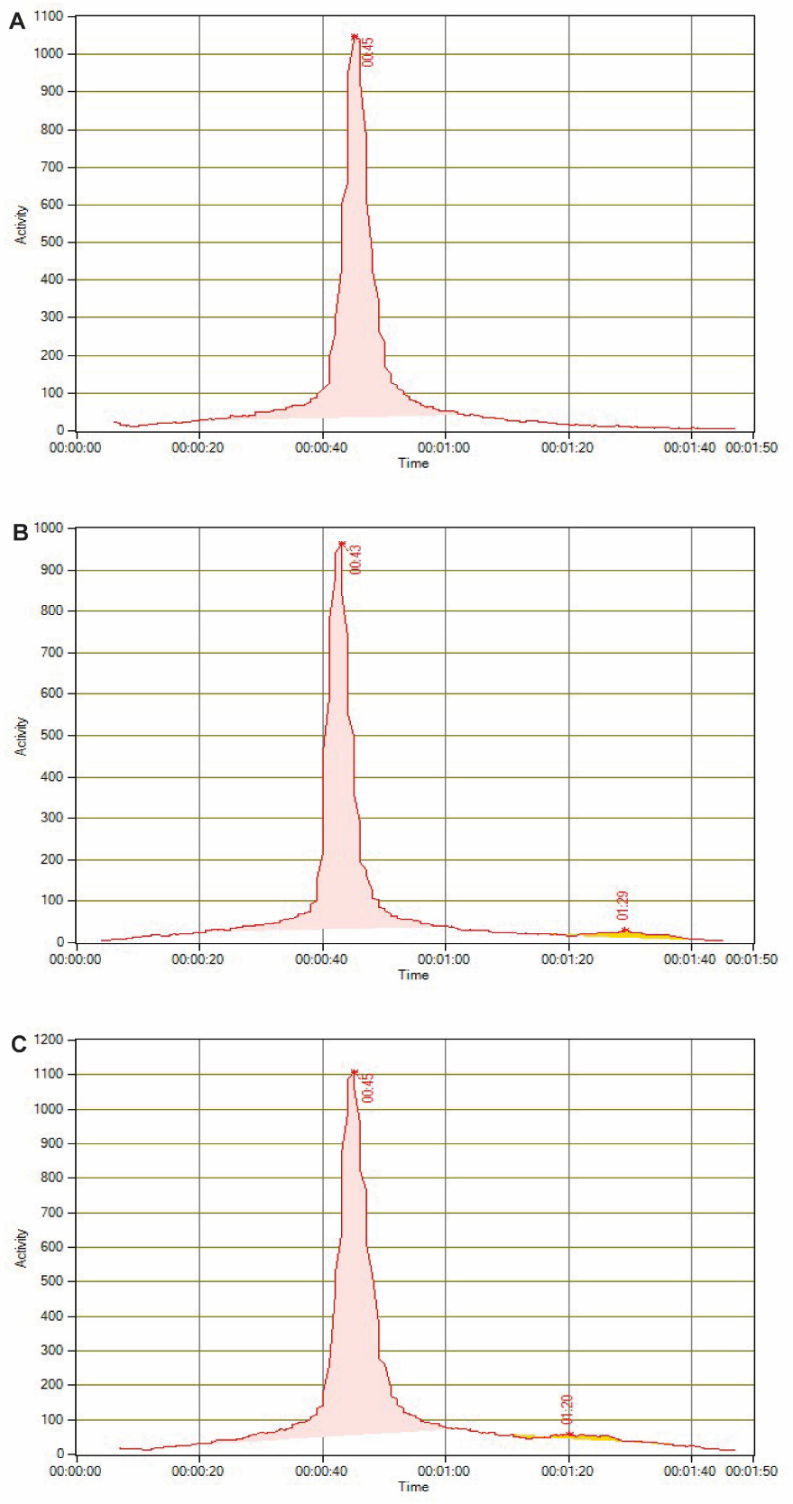


Figure S16.

The radiochemical purity of the three nanoparticles was analyzed prior to in vivo administration. (**A**) demonstrates 100% radiolabeling efficiency for FNP1. (**B**) 97% radiolabeling efficiency for FNP2 and (**C**) 98.85% radiolabeling efficiency for NFNP.

Biodistribution of nanoparticles


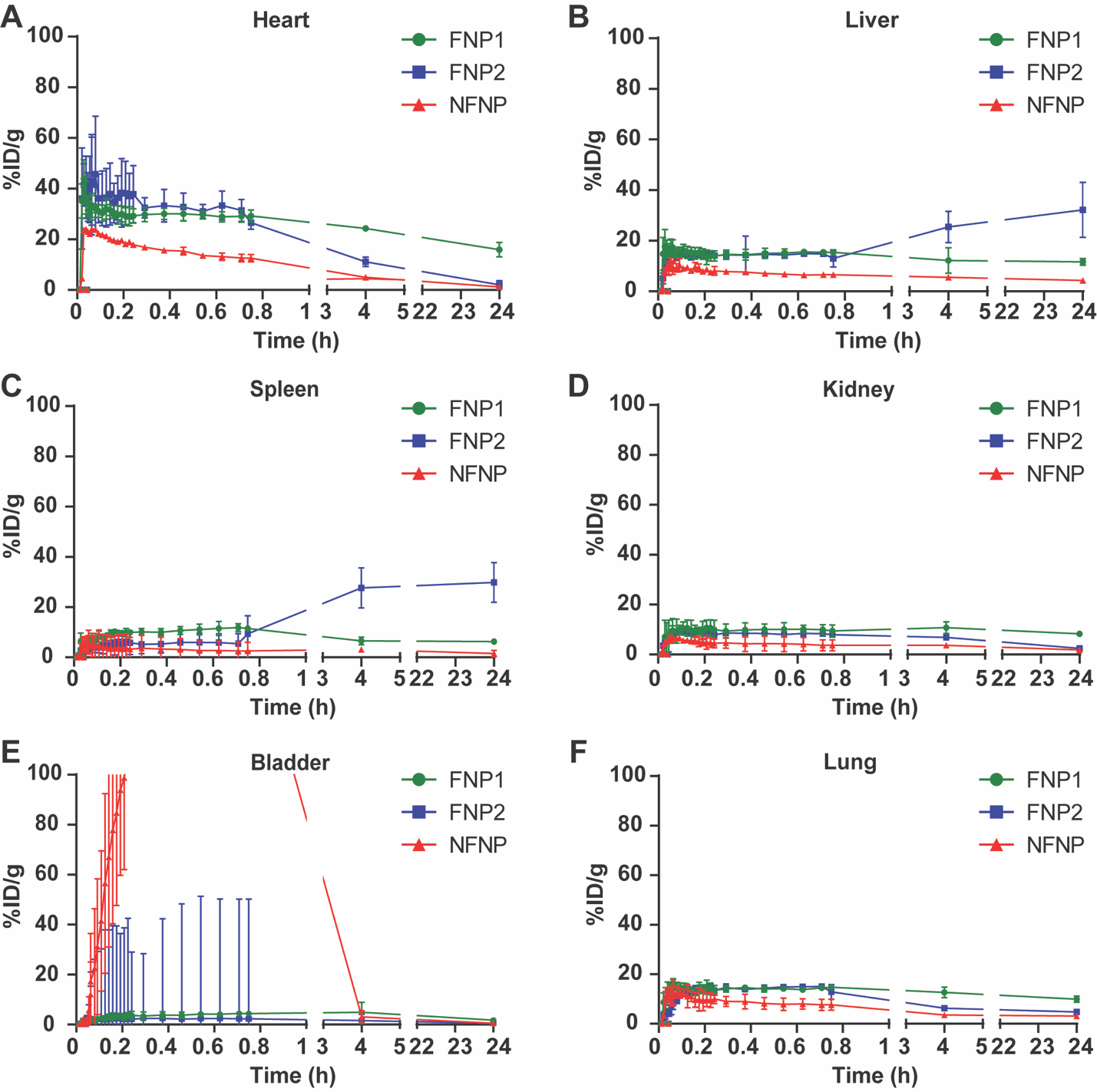


Figure S17.

Time Activity curves were derived from 3-dimensional volumetric regions of interest analysis of the temporal PET signal, guided by CT anatomical imaging. Counts per voxel were converted to becquerel per millilitre (Bq/mL) calculated according to the mouse weight based on a known scanner efficiency calibrated standard. Percentage of injected dose per gram (%ID/g) was further calculated over 24 hours. ^89^Zr radiolabelled nanoparticles FNP1 (green) FNP2 (blue) and NFNP (Red), are seen perfusing organs and clear from blood pool minutes following bolus injection (**A**) Heart, (**B**) Liver, (**C**) Spleen, (**D**) Kidney, (**E**) Bladder and (**F**) Lung were collected and counted at 25 hours post injection.


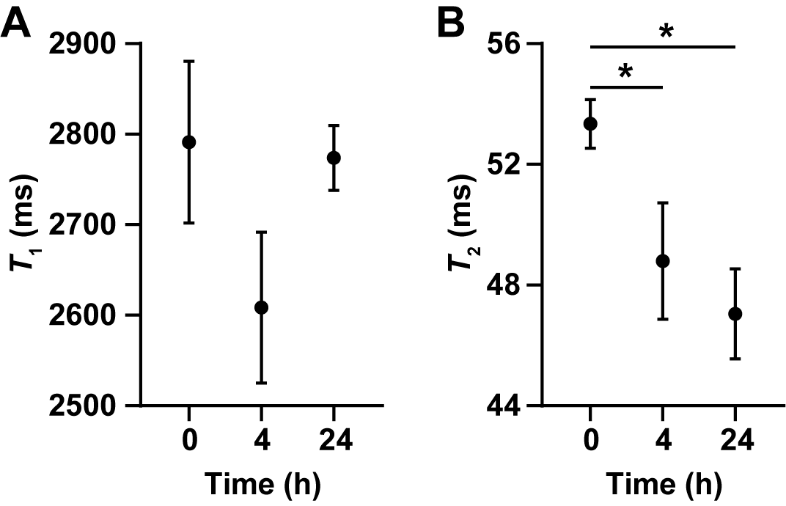


Figure S18.

MR imaging of a tumorous brain section of mice bearing medulloblastoma acquired before and at different time points after intravenous injection of 10 mg/kg FNP1. (**A**) *T*_1_ values and (**B**) *T*_2_ values extracted from the tumor sites, *p ≤ 0.05, two-way ANOVA, Tukey’s multiple comparisons test.


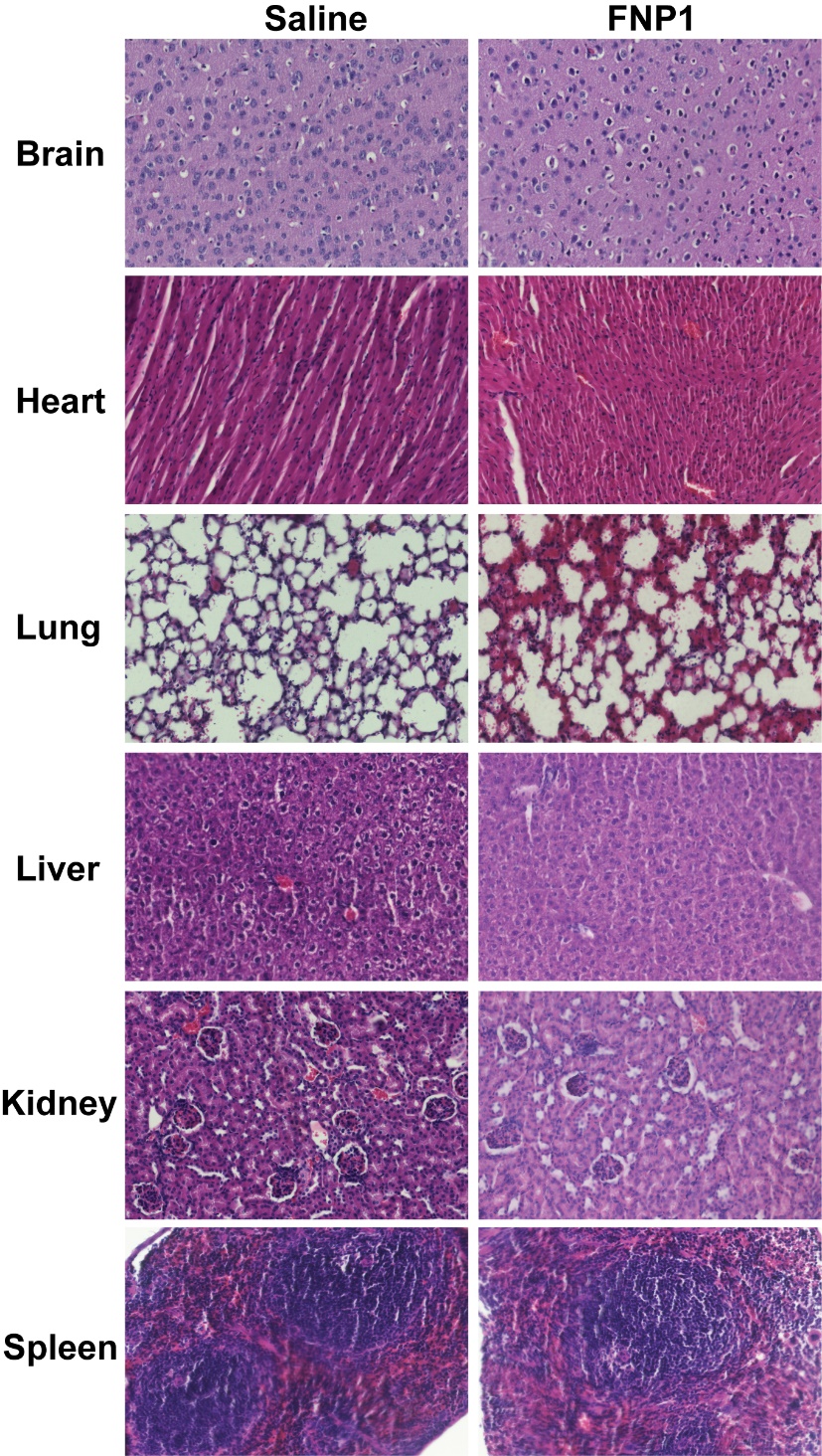


Figure S19.

H&E staining of tissues from major organs of mice after IV injected FNP1, comparing to the mice injected with PBS (20× magnification, 0.323 µm/pixel).

**Ethical approval for animal experiments**

For PET-CT imaging and *ex vivo* tissue analysis for biodistribution, all studies were in accordance with guidelines of the Animal Ethics Committee of The University of Queensland (UQ; Approval 2020/AE000044) and Australian Code for the Care and Use of Animals for Scientific Purposes.

The orthotopic medulloblastoma model was built in accordance with guidelines of the Animal Ethics Committee of The University of Queensland (UQ; Approval 2021/AE000666) and Australian Code for the Care and Use of Animals for Scientific Purposes.
